# A factorial design-optimized microfluidic LNP vaccine elicits potent magnesium-adjuvating cancer immunotherapy

**DOI:** 10.1016/j.mtbio.2025.101703

**Published:** 2025-03-24

**Authors:** Yongyi Xie, Jiaxin Guo, Jialin Hu, Yuan Li, Zhongqian Zhang, Yongcheng Zhu, Fei Deng, Jialong Qi, You Zhou, Wenjie Chen

**Affiliations:** a Guangzhou Municipal and Guangdong Provincial Key Laboratory of Molecular Target & Clinical Pharmacology, the NMPA and State Key Laboratory of Respiratory Disease, School of Pharmaceutical Sciences, Guangzhou Medical University, Guangzhou, 511436, PR China; b Department of Emergency, The Second Affiliated Hospital, Guangzhou Medical University, Guangzhou, 510260, PR China; c Yunnan Digestive Endoscopy Clinical Medical Center, Department of Gastroenterology, The First People's Hospital of Yunnan Province, Affiliated Hospital of Kunming University of Science and Technology, Kunming, 650032, PR China; d Graduate School of Biomedical Engineering, ARC Centre of Excellence in Nanoscale Biophotonics, Faculty of Engineering, UNSW Sydney, NSW, 2052, Australia

**Keywords:** LNP, Magnesium adjuvant, Microfluid, Cancer vaccine, Factorial design

## Abstract

Human papillomavirus (HPV)-associated cancers remain a critical health challenge, prompting the development of effective therapeutic vaccines. This study presents a lipid nanoparticle (LNP)-based vaccine co-loading E7 antigen peptide and magnesium ions as the adjuvant. Microfluidic technology was employed to optimize LNP preparation and formulation, ensuring efficient co-delivery of antigen and adjuvant. Magnesium ions were chosen over conventional aluminum-based adjuvants, which often suffer from limited efficacy and adverse effects, particularly for cancer immunotherapy. Compared to aluminum, magnesium ions exhibited superior capabilities in enhancing T-cell activation and promoting cellular immune response. Mechanistic insights suggest that magnesium ions facilitate dendritic cell maturation and antigen presentation via a collagen-CD36 axis, contributing to the adjuvant activity of magnesium. Through design of experiments (DoE) optimization, the LNP formulation was tailored for enhanced encapsulation and stability, positioning it as a targeted system for immune activation. These findings support the promise of magnesium ions as effective and safer adjuvants in LNP-based vaccines, marking a potential advancement for therapeutic cancer vaccination.

## Graphical abstract

**Figure:**
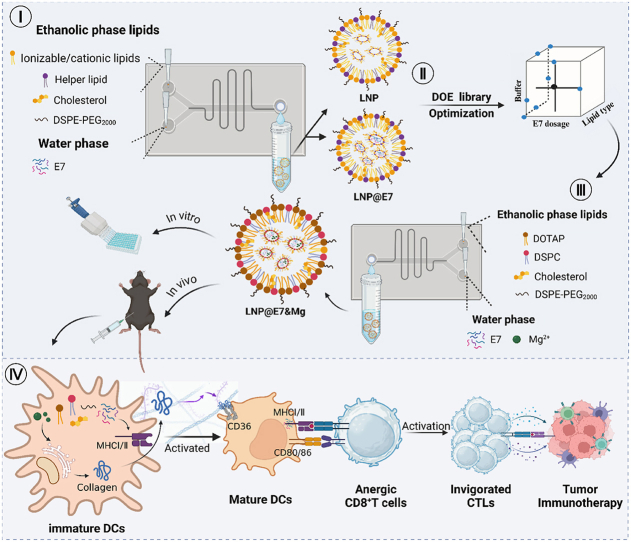

## 1 Introduction

Tumor immunotherapeutic including immune checkpoint inhibitors and cancer vaccines, is a promising strategy that aims to harness immune system to surveil and kill tumors. Among a range of immunotherapies, cancer vaccines have increasingly gained significant attention [1,2] due to their potency on cancer treatment. Unlike prophylactic vaccines that aim to prevent infections and occurrences, therapeutic vaccines are designed to inhibit or eradicate existing tumors by antigen induced specific cellular immune response [1]. Limited by the high complexity in preparation and the potential safety concerns of autologous extracted tumor antigens, current research primarily focuses on using synthesized amino acids- or nucleic acid-based antigens to induce anti-tumor immunity. Notably, vaccines formulated with subunit antigens have shown advantageous properties in less complexity, safety and stability [3,4]. For example, the E7 peptide derived from HPV (human papillomavirus) E7 protein, can activate cytotoxic T lymphocytes (CTLs) specific to cancer cells expressing the E7 antigen. Therapeutic vaccines targeting the E7 oncoprotein in HPV-related cancers have shown promise in preclinical studies [5,6]. For example, HPV16-SLP, a fusion E7 with E6 peptide antigen vaccine, has demonstrated the capability of long-term immunologic memory in low-grade abnormalities in the cervical cancer patients [7]. It could induce one-year lasting robust specific T cell response, which attributed to the clinical regression and viral clearance in several patients [8]. Despite the ongoing progress in cancer vaccines, the development of therapeutic vaccines yet confronts challenges such as insufficient immunogenicity, difficulties in precise targeting and cellular delivery of antigens, and the need for potent adjuvants to enhance immune responses [9]. For instance, the weak immunogenicity of peptide antigen alone and the lacking of self-adjuvant effects to stimulating innate immune responses led to the unsatisfied clinical outcomes. NEO-PV-01 is an impressive neoantigen vaccine consisting of up to 20 unique peptides into an integral formulation. Full NEO-PV-01 course has observed inducing neoantigen-specific CD4^+^ and CD8^+^ T cell responses in tumor patients, but rare clinical benefits can be found when comparing the patient's progression-free survival and overall survival [10]. All these necessitating the implements of effective adjuvants and delivery systems to enhance the immunogenicity and therapeutic efficacy of cancer vaccines.

Adjuvants play a crucial role in enhancing immune response, especially in the epitope-based therapeutic vaccines those requiring robust activation of the immune system [11]. One of the most widely used adjuvants in commercial is aluminum-based (alum) adjuvant, i.e. aluminum salts, including aluminum hydroxide and aluminum phosphate, have been used for decades to improve the immunogenicity of vaccines [12,13]. It works primarily by creating a depot effect, inducing the production of pro-inflammatory cytokines, and promoting the recruitment of antigen-presenting cells (APCs) particularly dendritic cells (DCs) at the injection site [14]. Despite their widespread applications, alum adjuvants primarily elicit a Th2-type immune response while inability to induce a strong Th1 response, which limits its effectiveness in vaccines designed to elicit a robust cytotoxic T-cell responses that required for cancer therapy [15]. Moreover, aluminum adjuvants could not induce significant mucosal immunity, which is critical for targeting tumors within mucosal tissues, such as in HPV-associated cancers. There are also concerns on the potential local and systemic adverse effects of alum, including inflammation and granuloma formation at the injection site [16,17]. This underscores the need for developing new adjuvants that can induce a stronger immune response with improved safety profiles.

Magnesium ions (Mg^2+^) have previously been applied as the stabilizer in vaccine components [18,19], or exert adjuvant therapy effect as the form of magnesium isoglycyrrhizinate for anti-inflammation, anti-oxidation and anti-virus applications [20]. However, its unique function as a novel candidate for vaccine adjuvant development has yet to be explored systematically. As far as we know, only one study utilized Mg^2+^ in a dule-adjuvants system for melanoma vaccine with the assistance of imiquimod to form a metal-complex [21]. Mg^2+^ plays a critical role in innate immunity and is involved in modulating immune cell functions [22,23]. It is known that magnesium deficiency in rodents can lead to various complications including eosinophilia, elevated IgE levels, and thymic atrophy [24,25]. While elevated magnesium ions promoted the recruitment and polarization of monocytes and macrophages during the early stage of bone formation after fractures [26]. Recent studies have highlighted magnesium's capacity of influencing tumor immunity, particularly by modulating the specific T cell activity. Mg^2+^ can enhance T-cell receptor (TCR) signaling, promoting the activation and proliferation of T cells [27]. Furthermore, Mg^2+^ is involved in the stabilization of cell membranes, which could aid in the efficient delivery of antigens to target cells. These characteristics, combined with the biocompatibility and natural abundance in the body, enable it an attractive alternative over traditional adjuvants [28]. However, the potential applications of magnesium ions as an adjuvant in cancer vaccines has not been extensively explored.

The development of lipid nanoparticle (LNP) delivery systems has revolutionized the field of vaccine and drug delivery. LNPs can encapsulate a variety of therapeutic agents, including nucleic acids, peptides, and small molecules, and protect them from enzymatic degradation while enhancing cellular uptake [29]. In the context of cancer vaccines, LNPs have been used to co-deliver antigens and adjuvants, enabling targeted delivery to APCs and facilitating the induction of a strong immune response [30]. However, to the best of our knowledge, very few studies on LNPs simultaneously carrying antigenic peptides and metal ion-based adjuvants such as magnesium were reported previously. Compared to conventional bulk mixing methods, microfluidic technology has further advanced the preparation of LNPs. By fine-tuning of parameters such as flow rate, lipid concentration, and solvent composition, it provides reproducible and precise control over particle size, composition, and encapsulation efficiency. The microfluidic synthesis of LNPs allows for the rapid preparation of uniformed nanoparticles with improved stability and delivery efficiency [31]. While recent studies have used microfluidics for optimizing LNPs in biomacromolecules delivery, including those focusing on nucleic acids delivery [32,33], our study adds a new dimension by incorporating a factorial design optimization (DoE) approach to better understand and control the interplay between parameters in Mg-adjuvanted LNP formulations. This systematic optimization enables a more precise tailoring of the LNP characteristics (such as size, encapsulation, and bioavailability) for the delivery of peptide/Mg-based cancer vaccines, a strategy not previously explored. This level of control is particularly important when co-loading antigens and adjuvants into a single delivery vehicle, as it ensures consistent dosing and bioavailability. In cancer immunotherapy, microfluidic-generated LNPs have shown promise in delivering mRNA vaccines, peptides, and other immunomodulatory agents with high precision and efficacy [34].

**Fig. 1:**
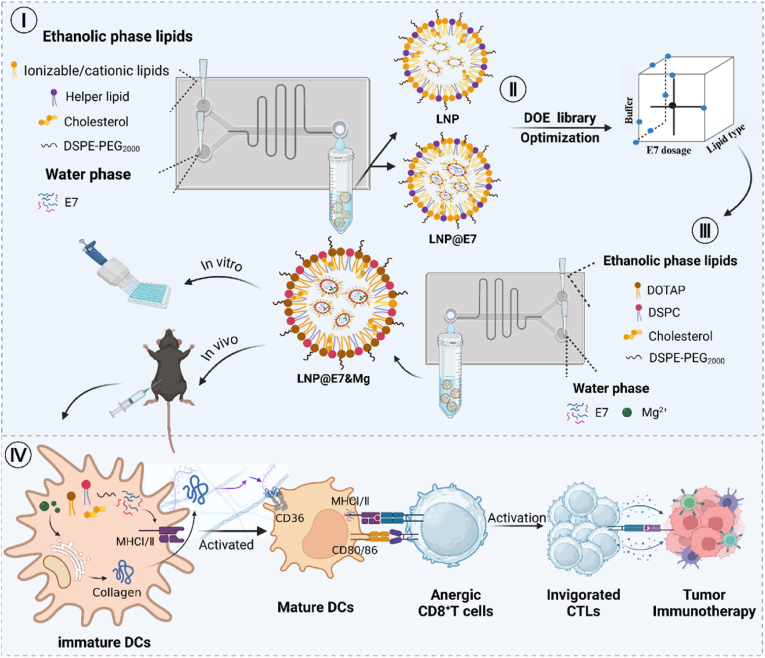
The scheme of this study illustrating the microfluid optimized LNP cancer vaccine adjuvating with magnesium. (I) Definitive screening design (DSD) to determine the lipid concentrations, FRR, and TFR in LNP preparation; (II) DoE of lipid type, buffer system, and E7 dosage; (III) Mg^2+^ incorporation to formulate the LNP@E7&Mg nanovaccine for following tests; (IV) Mechanism study of the adjuvanticity of magnesium and antitumor immunotherapy of the LNP@E7&Mg vaccine. Created via BioRender with permit ID: i61f781.

In this study, illustrated by Fig. 1, microfluidic technology was employed to prepare an LNP vaccine formulation co-loaded with the E7 antigen peptide and magnesium ions. This represents the first attempt to co-deliver a tumor antigen and a magnesium-based adjuvant using microfluid-fabricated LNPs. The factorial design approach was used to efficiently elucidate the relationships between multiple factors and LNP property variables while minimizing the times of tests. The co-delivery LNP formulation within the microfluidic synthesis process was optimized by systematically evaluating critical parameters including the total flow rate (TFR), flow rate ratio (FRR), buffer solutions, cationic lipid components, and the feeding of cargo molecule. Two independent experiments (3^3^ and 3^3^) were conducted to ensure the efficient encapsulation and enhanced delivery of both components. Additionally, we explored the possible mechanisms through which magnesium ions may act as an adjuvant, aiming to provide insights into their potential application in cancer immunotherapy. This delivery system holds promise for clinical application, offering a new pathway for developing more effective therapeutic vaccines for HPV-associated cancers with a novel adjuvating strategy.

## 2 Methods

### 2.1 Materials

Distearoyl Phosphatidylcholine (DSPC), (2,3-Dioleoyloxy-propyl)-trimethylammonium-chloride (DOTAP), DLin-MC3-DMA (Dlin-MC3), Cholesterol (Chol), DSPE-PEG2000 were purchased from AVT (Shanghai) Pharmaceutical Tech Co., Ltd. DODAP was produced from Shanghai Yuanye Bio Ltd. E7 peptide HPV16E7_44-62_ (_44_QAEPDRAHYNIVTFCCKCD_62_) was purchased from GL Biochem Shanghai Ltd., Cell Counting Kit-8 (CCK8 kit) was acquired from Glpbio (GK10001, USA). Unless otherwise specified, all chemicals were obtained from commercial sources and used without further purification. DMEM medium, RPMI 1640 medium, penicillin/streptomycin (P/S), 0.25 % trypsin-EDTA, phosphate buffered saline (PBS) and fetal bovine serum (FBS) were purchased from Gibco (USA).

### 2.2 Mice and cell lines

All cell lines were purchased from American Type Culture Collection (ATCC, USA). C57BL/6 mice (female, 4–6 weeks old) were obtained from the Guangdong Provincial Experimental Animal Center (Guangzhou, China). All the mice were raised in Specific Pathogen Free (SPF) animal facilities. All animal experiments were conducted in accordance with the guidelines approved by Guangzhou Medical University under the ethics approval GY2022-200.

### 2.3 Optimization of LNP-loaded E7 design using factorial design

Three key parameters were selected, each with three levels: lipid type (DOTAP, DODAP, and Dlin-MC3), buffer system (HEPES (25 mM), DEPC (0.1 %), and citric acid (25 mM)), and E7 dosage (0.05–0.2 mg). Experiments were conducted under all possible combinations (27 in total) to evaluate the impact of these factors on the loading efficiency of E7 in LNP. The data including key metrics such as average particle size, polydispersity index (PDI), and surface potential, were recorded and analyzed. The JMP pro software (Version 17) was used for DoE assay, ANOVA was then applied to the experimental data to identify main and interaction effects, determining how each variable influences the formation of LNPs and the encapsulation efficiency of E7.

### 2.4 Preparation and characterization of Mg^2+^ incorporated LNP vaccine

After the above DoE optimizations, LNPs loaded with E7 and Mg^2+^ were prepared using microfluidic technology with the optimized formulation. The organic phase consisted of DOTAP/Chol/DSPC/DSPE-PEG2000 (molar ratio of 50/10/39/1) in an ethanol solution, with a total lipid concentration of 5 mg/mL. E7 and magnesium chloride were dissolved in DEPC (0.1 %) water as the aqueous phase. These two solutions were mixed using a staggered herringbone micromixer at a total flow rate (TFR) of 1.2–3.2 mL/min, with a water/ethanol flow rate ratio (FRR) of 3:1. The sample collected at the outlet of the micromixer and dialyzed overnight in approximately 1000 times of DPBS (pH 7.4) solution using a dialysis bag with a molecular weight cut-off of 3000 Da. The mixture was then ultrafiltered and centrifuged at 8000 rpm for 20 min. The final volume was adjusted with DPBS to obtain the desired concentration, resulting in the LNP samples.

The morphology and the element mapping of the Mg^2+^ incorporated LNP vaccine was characterized using a JEM-1400PLUS transmission electron microscope (TEM), and dynamic light scattering measurements were performed using a Malvern zetasizer (Nano-ZS90). The encapsulation efficiency of E7 and Mg^2+^ were determined using the bicinchoninic acid (BCA) assay (20201ES76, Yeasen Bio.) and inductively coupled plasma mass spectrometry (ICP-MS), respectively.

### 2.5 In vitro cellular uptake of Mg^2+^ incorporated LNP- peptide vaccine

The in vitro cellular uptake was investigated using confocal laser scanning microscopy (CLSM) and flow cytometry. DC2.4 cells were cultured in glass-bottom dishes at a cell density of 1 × 10^4^ cells. Free E7 (E7-FAM), LNP@E7-FAM, or LNP@E7-FAM&Mg were then added to the cells. After 24 h of incubation, the cells were stained with DAPI for 10 min. Imaging was performed using a Leica DLS microscope to visualize cellular uptake. For flow cytometry, DC2.4 cells were seeded into 6-well plates, and then treated with FAM-E7 just as in the CLSM experiment. After 24 h incubation, the cells were washed twice for flow cytometry using CytoFLEX Flow Cytometer from Beckman Coulter Life Sciences. For endosomal tracking of the LNP vaccines, lysosomes were labeled with lysotraocker and the FAM labeled E7 were used to trafficking dynamically after various time of incubation, imageJ was used to profiling the colocalization between two channels and the PCC (Pearson's correlation coefficient) was calculated.

### 2.6 Biosafety assessment of LNP vaccines

For cell viability assay, DC2.4 cells were seeded into 96-well plates at a density of 1 × 10^5^ cells per well. The cells were then incubated with different concentrations of LNP@E7&Mg nanovaccine for 24 h. Finally, cell viability was assessed using a CCK8 assay kit. For hemolysis assay, the whole blood was collected from healthy mice by eyeball enucleation and stored in anticoagulant tubes containing sodium citrate. After gentle mixing, the blood was centrifuged at 3000 rpm for 15 min to isolate the red blood cells. A volume of 20 μL of the isolated red blood cells was then mixed with 1 mL of different concentrations of the sample nanovaccines (25–200 μg/mL), 1 mL of ddH_2_O, or 1 mL of PBS solution. The mixtures were incubated at 37 °C for 4 h, followed by centrifugation at 2000 rpm for 20 min. Subsequently, 100 μL of the supernatant from each sample was transferred to a 96-well plate, and absorbance at 542 nm was measured using a microplate reader. Each measurement was conducted with three replicates, and the standard deviation (SD) was calculated. The hemolysis rate was determined using the following formula: Hemolysis rate = (OD(sample) - OD(PBS group))/(OD(ddH_2_O) - OD(PBS group)∗100 %.

### 2.7 The activation assays of bone marrow-derived dendritic cells (BMDCs)

Bone marrow-derived dendritic cells (BMDCs) were isolated from the tibia and fibula of female C57BL/6 mice. The process began by removing the muscles from the legs to expose the intact bones, which were then soaked in 75 % ethanol. Using sterilized tools, one end of each bone was cut open, and a disposable syringe filled with 1640 complete culture medium was inserted to flush the bone marrow into a centrifuge tube. The resulting cell suspension was gently mixed and centrifuged at 1500 rpm for 5 min, followed by the lysis of red blood cells with a lysis buffer. The cell suspension was transferred to a non-tissue-treated dish and supplemented with 20 ng/mL mouse granulocyte-macrophage colony-stimulating factor (GM-CSF). The mixture was then gently placed in an incubator for static culture. The culture medium was carefully replaced with fresh complete medium every 2 days. On the 8th day, semi-suspended dendritic cells were collected, yielding the bone marrow-derived dendritic cells (BMDCs).

The activation of BMDCs by different materials was analyzed by assessing cytokine production and the expression of surface markers. BMDCs were incubated with PBS, E7, LNP@E7, E7&Mg, LNP@E7&Al, or LNP@E7&Mg for 24 h. The concentration of cytokines in the supernatant was measured using enzyme-linked immunosorbent assay (ELISA) kits provided by Elabscience Ltd. Flow cytometry was performed using Pcy5.5-CD11c, PE-CD80, and APC-CD86 antibodies to analyze the expression of surface molecules on BMDCs. Data were collected using a CytoFLEX flow cytometer (Beckman, USA) and analyzed with FlowJo Software 10.0 (USA).

### 2.8 Transcriptome sequencing analysis

BMDCs were seeded in 6-well plates and treated with PBS, LNP@E7, LNP@E7&Al, or LNP@E7&Mg for 24 h. After treatment, the culture medium was removed, and the wells were washed twice with enzyme-free PBS. Following this, 1 mL of Trizol lysis solution (15596018, Invitrogen, USA) was added to each well. BMDCs were lysed in Trizol, and the cells were thoroughly pipetted to ensure complete detachment. The cell lysate was then collected into cryogenic tubes, rapidly frozen in liquid nitrogen, and stored at −80 °C after which the mRNA was purified from the total RNA (5 μg) using Dynabeads Oligo (dT) (Thermo Fisher, USA) through two rounds of purification. The Magnesium RNA Fragmentation Module (e6150, NEB, USA) was employed at 94 °C for 5–7 min to fragment the mRNA into short fragments, and the cDNA was synthesized using SuperScript™ II Reverse Transcriptase (1896649, Invitrogen, USA). The U-labeled second-stranded DNAs were synthesized using DNA polymerase I (m0209, NEB, USA), RNase H (m0297, NEB, USA), and dUTP Solution (R0133, Thermo Fisher, USA). Subsequently, an A-base was added to the fragmented DNA to ligate with the T-base overhang adapters, and dual-index adapters were then ligated to the fragments. AMPure XP beads were used for size selection. The U-labeled second-stranded DNAs were treated with a heat-labile UDG enzyme (m0280, NEB, USA), followed by a PCR procedure that included: I) denaturation for 3 min at 95 °C; II) 8 cycles of 15 s at 98 °C; III) annealing for 15 s at 60 °C; IV) extension for 30 s at 72 °C; and V) a final extension for 5 min at 72 °C. The final cDNA libraries had an average insert size of 300 ± 50 bp. The sequencing was completed with 2 × 150 bp paired-end sequencing (PE150) on an Illumina NovaSeq™ 6000 (LC-Bio Technology CO., Ltd., China).

### 2.9 In vivo study of the antitumor efficacy of nanovaccines

C57BL/6 mice were subcutaneously injected with 1 × 10^6^ TC-1 cells into the right flank. On day 7, the mice were randomly divided into six groups for various treatments: (1) PBS, (2) E7, (3) LNP@E7, (4) E7&Mg, (5) LNP@E7&Mg, and (6) LNP@E7&Al. The mice were then subcutaneously vaccinated on days 7, 11, 15, and 19. Tumor volume and body weight were monitored regularly, with tumor volume calculated as (length × width × width)/2. On day 21, the mice were sacrificed. The tumors, spleens, draining lymph nodes were collected for immunological analysis, while the blood and main organs were collected for biosafety assay.

### 2.10 Flow cytometry analysis of immune cell population

Fresh tumor tissues, lymph nodes, and spleens were collected and analyzed for antitumor immune responses using flow cytometry. Briefly, samples were dissociated into single-cell suspensions and red blood cells were removed using red blood cell lysis buffer (Solabio, China). The samples were then blocked in PBS containing 0.1 % BSA (36101 ES, Yeasen Bio.) and incubated with the relevant antibodies at room temperature for 1 h. To characterize immune cell populations, the following antibodies were used for staining: CD3-Pcy5.5 (F1013J, Elabscience), anti-mouse CD4-FITC (F1097C, Elabscience), anti-mouse CD8a-PE (F1104D, Elabscience), anti-mouse CD11b-FITC (F1081C, Elabscience), anti-mouse Gr-1-PE (F1120D, Elabscience), anti-mouse IFN-γ-APC (F1101E, Elabscience), anti-mouse Foxp3-PE (F1238D, Elabscience), anti-mouse CD62L-Pcy5.5 (F1011J, Elabscience), and anti-mouse CD44-APC (F1100E, Elabscience) (All antibody information was listed in Table S4). Specifically, cells were permeabilized in 0.2 % triton-X 100 for 10 min at room temperature during the intracellular cytokines staining procedure. Data were collected using a CytoFLEX flow cytometer (Beckman, USA) and analyzed with FlowJo Software (USA). The gating strategies were displayed in Figs. S12–14.

### 2.11 Immunohistochemistry and immunofluorescence

Hematoxylin and eosin (H&E) staining, along with TUNEL staining, was employed to observe histological changes in tumor tissue sections. For immunohistochemical (IHC) analysis of immune cell infiltration, tissue sections were incubated with anti-CD8 or anti-Foxp3 antibodies (Servicebio). Finally, images were captured using a fluorescence microscope.

### 2.12 Statistical analysis

All values are reported as the mean ± standard deviation (SD). Data were evaluated by on way analysis of variance (ANOVA) for comparison of multiple groups using GraphPad Prism 9.5 software.

## 3 Results and discussion

### 3.1 Optimization of the preparation of LNP@E7 nanovaccine

**Fig. 2:**
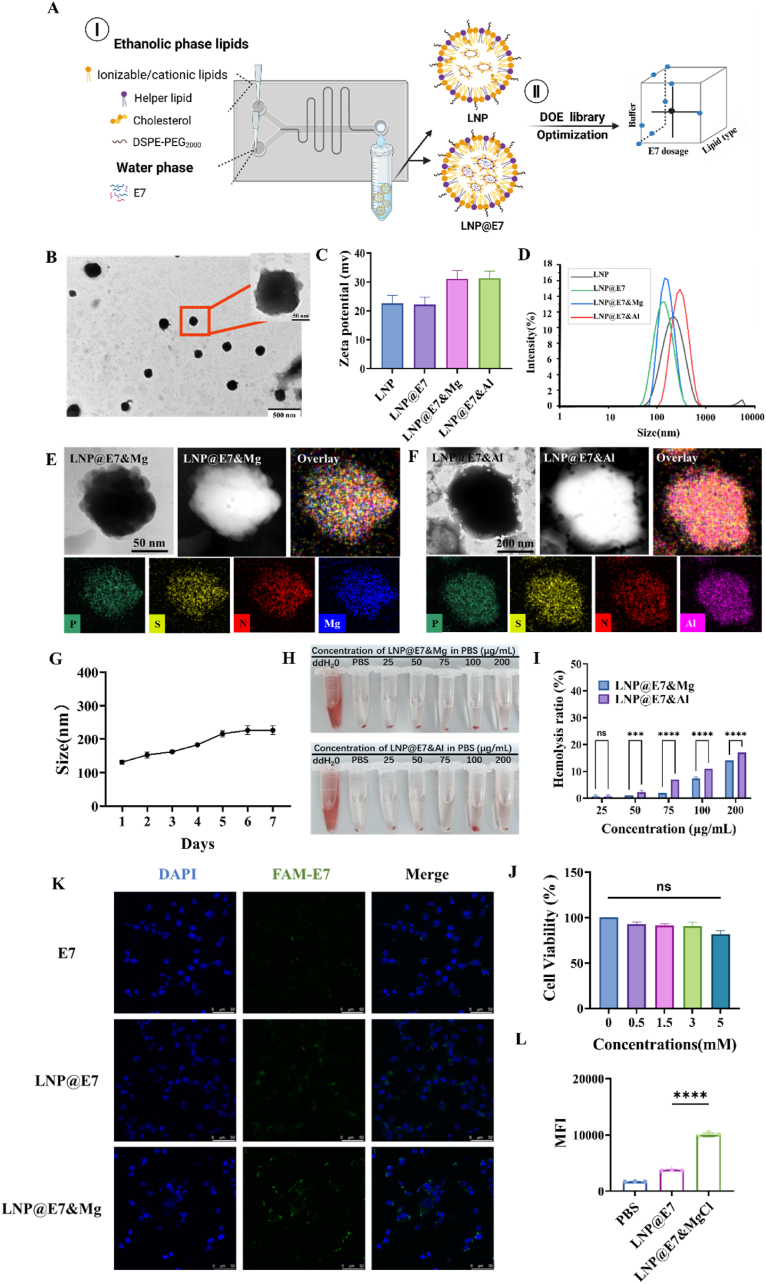
Characterization, cell viability, and cellular uptake of nanovaccines. (A) The diagram of microfluid-based LNP and LNP@E7 preparation optimized using DoE; (B) Representative TEM image of LNP@E7&Mg, scale bar is 500 nm in the inset picture; (C) Zeta potential analysis of LNP vaccine particles; (D) DLS profile of LNP@E7&Mg; (E, F) The representative images of mapping of LNP@E7&Mg and LNP@E7&Al; (G) The curve of size changes of LNP@E7&Mg over time; (H) Hemolysis assay of nanovaccines at different concentrations; (I) Statistical analysis of hemolysis at various concentrations of nanovaccines; (J) Cell viability assay for the safety of nanovaccines at different concentrations; (K) Confocal microscopy images of DC2.4 cells showing the cellular uptake of E7 peptide after 6 h of incubation, scale bar: 50 μm; (L) The median fluorescence intensity (MFI) in the flow cytometry assay of the cellular uptake of DC2.4 cells by different treatments of nanovaccines for 6 h ∗p < 0.05, ∗∗p < 0.01, ∗∗∗p < 0.001, ∗∗∗∗p < 0.0001, was considered significantly different between compared groups using ANOVA test.

Design of experiments were performed to obtain the optimum co-delivering-LNP formulation synthesis procedure in microfluidic device (Fig. 1A). First, the study established a specific lipid molar concentration formulation of DOTAP/Chol/DSPC/DSPE-PEG_2000_ (molar ratio of 50/10/39/1) to test the effects of various parameters on the size and dispersion of LNP particles. Prior to optimize the formulation parameters in LNP preparation, FRR and TFR were found to have significant impacts, particularly the interaction between FRR and TFR (TFR∗FRR) which significantly influenced particle size (p = 0.0118) (Fig. S1). Specifically, the total flow rate (TFR) was varied from 1.2 to 3.2 mL/min, the flow rate ratio (FRR) from 3 to 9, and the total lipid concentration from 1 to 4 mM, with 14 runs conducted. Based on the particle size and PDI obtained from the tests, a multiple regression equation was constructed, indicating that these factors significantly influenced the response values. The optimal lipid concentration was determined to be 2.11 mM, with a TFR of 2.56 mL/min (Table S1). Subsequently, the data analysis using JMP Pro software with factorial design (Figs. S2–3) indicates lipid composition, buffer type, and E7 concentration were significantly influence the DLS results of LNP@E7 nanoparticles. Ultimately, using microfluidic technology to prepare LNPs loaded with E7 and magnesium ions, a 3 × 3 × 3 factorial experimental design was developed to optimize the co-loading of E7 and Mg^2+^ into LNPs. The experiment involved varying three key parameters: cationic or ionizable lipids (DOTAP, DODAP, and Dlin-MC3), buffer system (HEPES (25 mM), DEPC (0.1 %), and citric acid (25 mM)), and E7 dosage (0.05–0.2 mg) (Fig. 2A). The interactions among these parameters and their effects on the dynamic light scattering (DLS) and encapsulation efficiency (EE%) of LNP were analyzed to identify the most efficient LNP carrier for co-loading E7 and Mg^2+^. According to the design created by JMP Pro software, a total of 27 different LNP formulations were prepared. These 27 LNP samples loaded with E7 (LNP@E7) were produced using microfluidics in sequential order, and their DLS and EE% were subsequently measured. The results were then entered into the factorial design plan for statistical analysis (Table S2). Based on the data analysis performed with JMP Pro software, it was determined that when the buffer system was DEPC (0.1 %) and the lipid type was DOTAP, the LNP@E7 exhibited the smallest particle size, the lowest PDI, and the highest EE% (Figures S4 A-B). Since the particle size of nanovaccine would influence the uptake of the vaccine components by APCs in vivo, small-sized nanoparticles are beneficial for subsequent T cell activation and inducing a stronger immune response, the smallest LNP with highest antigen loading was selected for following experiments in this study. In conclusion, DEPC (0.1 %) buffer and DOTAP were selected for the preparation of LNP in following studies.

### 3.2 Preparation and characterization of LNP@E7&Mg^2+^

LNPs co-loaded with the antigen E7 and adjuvant Mg^2+^ (LNP@E7&Mg) were prepared following the method described above (Fig. 2A). For comparison with commercially available adjuvants, LNPs loaded with the antigen E7 and adjuvant Al^3+^ (LNP@E7&Al) were also prepared. Transmission electron microscopy (TEM) revealed that LNP@E7&Mg has a uniform spherical morphology (Fig. 2B), and the Mg^2+^ ions and Al^3+^ ions are doped in the internal cavity of the LNP (Fig. 2E and F). The zeta potential of LNP@E7&Mg was approximately 30 mV (Fig. 2C). The DLS analysis showed that as-prepared LNP@E7&Mg has an average particle size of 125 ± 1.1 nm and a PDI of 0.13 ± 0.08 (Fig. 2D), and it remained relatively stable over 7 days (Fig. 2G). The encapsulation efficiency of E7 was 89.51 ± 0.4 %, with a drug loading capacity of 1.28 ± 0.06 %. The encapsulation efficiency of Mg^2+^ was 15.52 ± 0.1 %, with a loading capacity of 2.59 ± 0.3 % (Table S3). The release curves of Mg^2+^ and E7 in LNP@Mg&E7 in PBS with pH = 7.4 over 72 h are shown in Fig. S5, indicating a rapid release within 0.5 h and thereafter reaching a plateau of release of both antigen and Mg at 24 h.

Neither LNP@E7&Mg nor LNP@E7&Al vaccines induced significant hemolysis at various concentrations, but LNP@E7&Al shows higher hemolysis in the same concentrations, indicating lower potential toxicity and better safety of LNP@E7&Mg, in contrast to the Al adjuvanted LNP vaccine (Fig. 2H and I), which may be contributed by the various mechanisms of interaction with lipid bilayer and RBCs [35,36], and the instability of Al^3+^-loaded LNP [37]. After incubating with different concentrations of LNP@E7&Mg with DC2.4 cells for 24 h, cell viability was assessed (Fig. 2J). The results indicated that LNP@E7&Mg did not significantly affect the viability of DC2.4 cells at concentrations below 5 mM. Considering the balance between toxicity and the required dose of the E7 antigen, a nanovaccine concentration of 1.5 mM was selected for subsequent experiments. Furthermore, confocal microscopy results demonstrated that LNP@E7&Mg enhances cellular uptake, which may be contributed by the improved particle stability as well as the increased zeta potential (Fig. 2C) of LNP after the addition of Mg^2+^, suggesting that Mg^2+^ in LNP encapsulation can remarkably facilitates cellular internalization of LNPs and the vaccine delivery into cells (Fig. 2K–L and Fig. S6). In addition, Mg^2+^ has demonstrated to promote the endosomal escape of E7 peptide after 12 h of incubation of LNP@E7-FAM&Mg, showing the lowest PCC value (PCC = 0.56, Fig. S9) compared to the E7-FAM (PCC = 0.77, Fig. S7) and LNP@E7-FAM treated group (PCC = 0.67, Fig. S8).

### 3.3 LNP@E7&Mg promotes activation and maturation of BMDCs in vitro

**Fig. 3:**
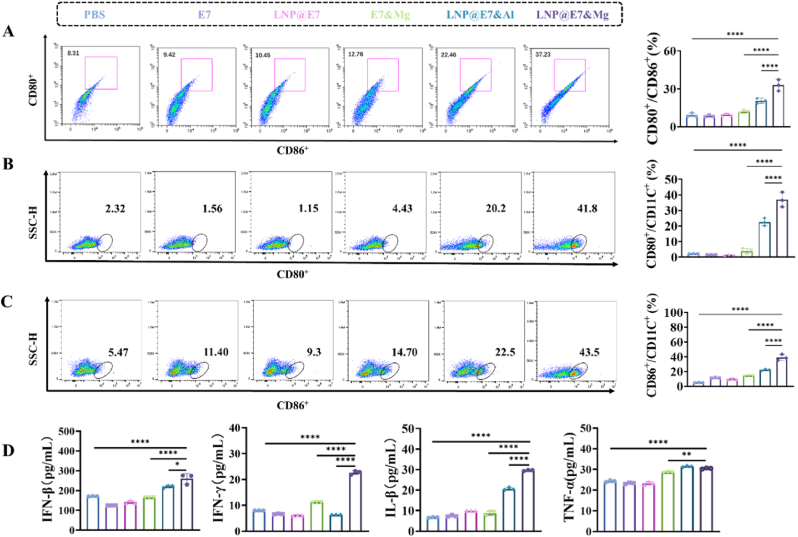
Activation of BMDCs in vitro. (A) Analysis of CD80 CD86 populations expression on the surface of BMDCs treated with nanovaccines and corresponding statistical graph; (B) Analysis of CD80 expression on the surface of BMDCs treated with nanovaccines and corresponding statistical graph; (C) Analysis of CD86 expression on the surface of BMDCs treated with nanovaccines and corresponding statistical graph; (D) Secretion levels of IFN-β, IFN-γ, IL-1β, and TNF-α of BMDCs after treatment with nanovaccines. ∗p < 0.05, ∗∗p < 0.01, ∗∗∗p < 0.001, ∗∗∗∗p < 0.0001, was considered significantly different between compared groups using ANOVA test.

After 24 h of incubation with PBS, free E7, free E7&Mg^2+^, LNP@E7, LNP@E7&Al, or LNP@E7&Mg, the ability of LNP@E7&Mg to stimulate the activation and maturation of bone marrow-derived dendritic cells (BMDCs) was assessed by flow cytometry, analyzing the expression of cell maturation markers CD80 and CD86. The gating strategy for cell populations is shown in Fig. S10, and the results indicated that LNP@E7&Mg significantly enhanced the expression of CD80 and CD86, demonstrating enhanced activation of BMDCs compared to other treatments (Fig. 3A–C). Moreover, the results showed that Mg^2+^, as an adjuvant in the LNP nanovaccine, outperformed Al^3+^ in effectively stimulating BMDCs which may consequently trigger a stronger immune response. Additionally, cytokine levels in the BMDC supernatants were measured by ELISA, revealing that LNP@E7&Mg promoted the secretion of TNF-α, IL-1β, IFN-γ, and IFN-β (Fig. 3D). This suggests that the inclusion of Mg^2+^as an adjuvant could induce higher levels of cytokine secretion, significantly enhancing the ability of LNP@E7&Mg to stimulate BMDC maturation. These findings indicate that the nanovaccine containing Mg^2+^ not only successfully loads and delivers the antigen but also can effectively activate APCs those are crucial in antigen presenting and initiating signal for stimulated immune response.

### 3.4 Mechanism study of the adjuvanticity of the Mg^2+^

**Fig. 4:**
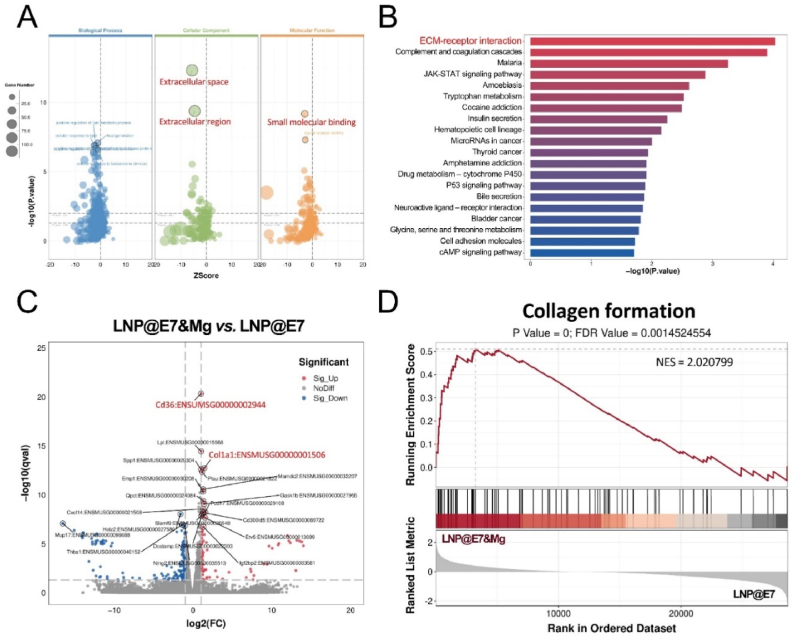
Mechanism study of the Mg^2+^ adjuvanticity by RNA-seq analysis (A) Bubble plot of GO enrichment analysis between LNP@E7&Mg and LNP@E7 treated BMDCs; (B) The top 20 enrichment of differently expressed genes in LNP@E7&Mg treated BMDCs compared to those treated with LNP@E7; (C) The volcano plot of gene regulation profile with the top two upregulated genes (Cd36, Col1a1) colored red; (D) GSEA analyzed enrichment of collagen formation in BMDCs treated with LNP@E7&Mg and LNP@E7. (For interpretation of the references to color in this figure legend, the reader is referred to the Web version of this article.)

To further explore the adjuvanticity mechanisms of the Mg^2+^ in nanovaccines, BMDCs were incubated with LNP@E7 and LNP@E7&Mg, followed by transcriptome sequencing and bioinformatic analysis. The adding of Mg^2+^ changed an abundant of gene expression, Gene Ontology (GO) enrichment analysis (Fig. 4A) indicated that LNP@E7&Mg mainly influences cellular component of extracellular space, extracellular region, and the molecular function of small molecular binding. While Kyoto encyclopedia of genes and genomes (KEGG) analysis result showed the most significant enrichment pathway is ECM-receptor interaction (Fig. 4B). To find out the specific potential genes that mediate the adjuvanticity of Mg^2+^, the gene expression fold change was calculated and ranked by *p* value, CD36 and Col1a1 were the top differently expressed genes (Fig. 4C and Fig. S11A). It is well known that CD36 is a widely expressed glycoproteins on immune cells interacts with collagen in extracellular matrix [38,39]. The stimulation of CD36 on DCs participated in the antigen presentation and T cell priming functions [40,41]. Whereas there were studies indicating that Mg^2+^ could promote the stabilization of collagen [42,43], which consistently demonstrate the underline mechanism of Mg^2+^ in DC maturation and antigen presentation. Gene Set Enrichment Analysis (GSEA) were further performed to determine the metabolism of collagen, and the results showed the hyperactive collagen formation (Fig. 4D). GSEA revealed that several genes associated with collagen synthesis and structure including Col1a1, Col3a1, Col1a2, and Col6a3 et al., were all significantly upregulated by the influence of Mg^2+^ in comparison to Al^3+^ -based adjuvant (Fig. S11B). This suggests that Mg^2+^ may actively enhance collagen gene expression within the local environment. Furthermore, GSEA identified a marked upregulation in collagen chain trimerization processes (Fig. S11C), indicating that Mg^2+^ facilitates the formation of stable collagen structures. These findings suggest a potential Mg^2+^-mediated collagen-CD36 signaling axis in DCs, supporting an enhanced maturation and antigen presentation process. The ability of Mg^2+^ to modulate collagen-related pathways within DCs could contribute significantly to its adjuvant effects in the LNP@E7&Mg vaccine formulation, enhancing the immune response and potentially offering advantages over traditional Al^3+^-based adjuvants.

### 3.5 Antitumor activity of LNP@E7&Mg vaccine

**Fig. 5:**
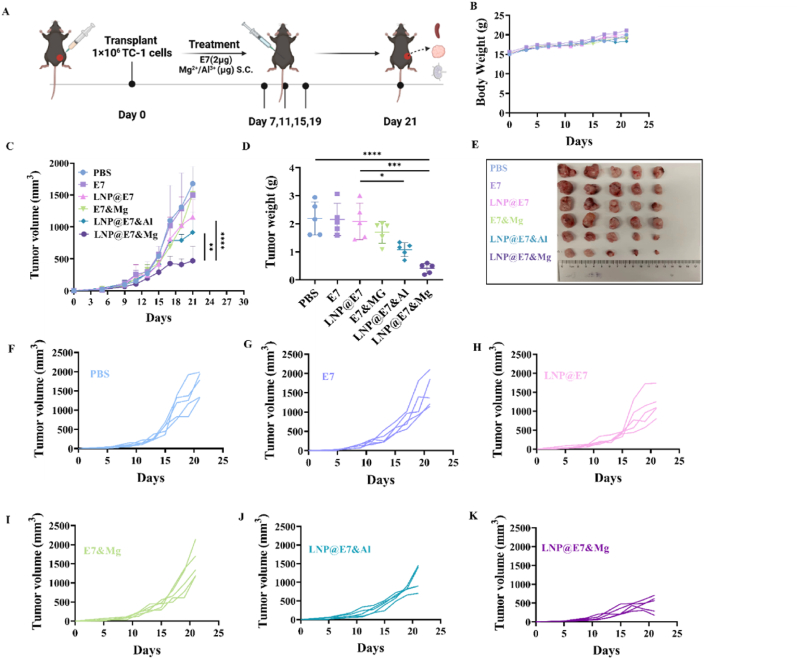
Antitumor activity of nanovaccines. (A) Immunization schedule of mice, n = 5; (B) Body weight growth of mice in each group; (C) Tumor growth of each group over 21 days; (D) Tumor weights of mice in each group; (E) The photographs of tumors; (F–K) Dynamic monitoring of tumor growth of single mice in each group. ∗p < 0.05, ∗∗p < 0.01, ∗∗∗p < 0.001, ∗∗∗∗p < 0.0001, was considered significantly different between compared groups using ANOVA test.

In this study, we established a subcutaneous TC-1 cell tumor model to evaluate the antitumor effects of the Mg^2+^ adjuvanted nanovaccine (Fig. 5A). During the therapeutic process, body weights of mice changed without any significance between groups with various treatments, indicated good biosafety profile of the LNP vaccines (Fig. 5B).Tumor growth was monitored (Fig. 5C), the tumors were weighed (Fig. 5D) and photographed (Fig. 5E) at the endpoint results, showing that LNP@E7&Mg vaccination led to a significantly more tumor suppression and less tumor weight compared to any other groups including the Alum-adjuvanted vaccination (LNP@E7&Al) (Fig. 5C and D). Analysis of tumor growth curves of each single mouse in all the groups further supported these findings (Fig. 5F–K). These results demonstrate that the Mg^2+^ adjuvanted LNP therapeutic vaccine exhibits the strongest antitumor activity during treatment.

### 3.6 Enhanced antitumor immune response induced by Mg^2+^ adjuvanted LNP vaccine

To evaluate the immunological impact of the Mg^2+^-adjuvanted LNP vaccine on antitumor response in mice, immune cell populations were examined across the spleen, lymph nodes, and tumor tissues using flow cytometry and immunohistochemical (IHC) analysis. Distinct changes of immune cell composition in each tissue help delineate the local and systemic responses facilitated by the Mg^2+^ adjuvant in comparison to control treatments.

**Fig. 6:**
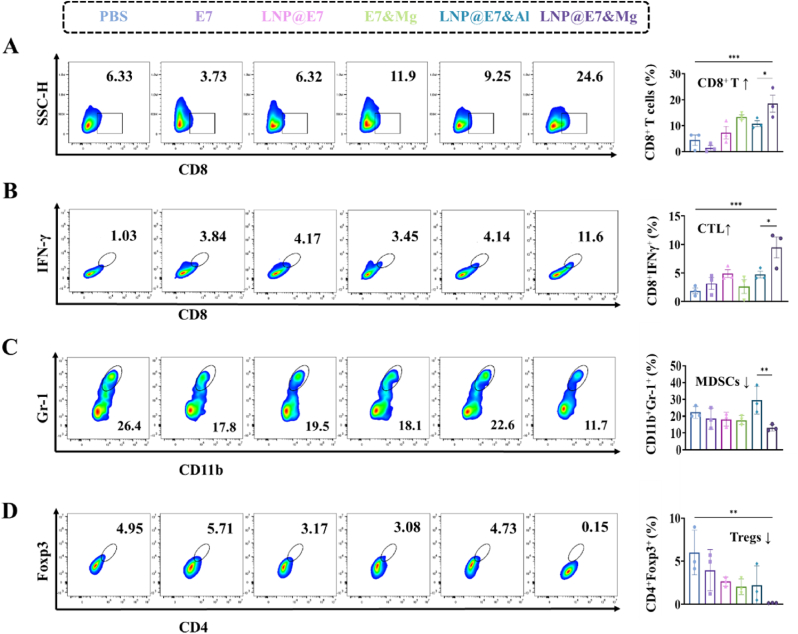
Flow cytometric analysis of immune cells in tumor tissues treated with different nanovaccines. (A) Flow cytometry analysis and statistical data showing the CD8^+^ T cell content in tumor tissues. (B) Representative images and statistical data of CTLs (CD8^+^IFNγ^+^) in tumor tissues. (C) Flow cytometry analysis and statistical data of MDSCs (CD11b^+^Gr-1^+^) in tumor tissues. (D) Representative flow cytometry plots and statistical data of tumor-infiltrating Tregs (CD4^+^Foxp3^+^) in tumor tissues. ∗p < 0.05, ∗∗p < 0.01, ∗∗∗p < 0.001, was considered significantly different between compared groups using ANOVA test.

**Fig. 7:**
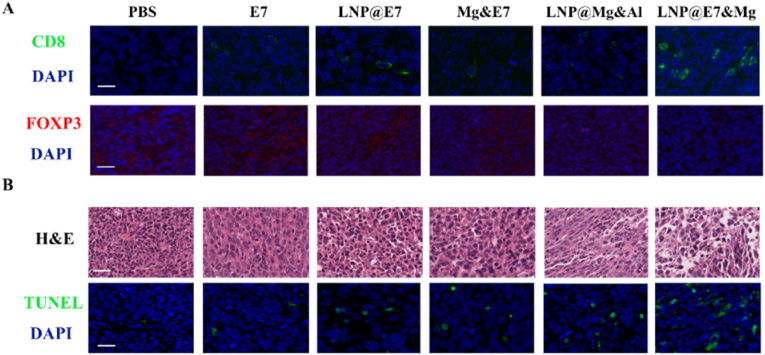
Immunohistochemical analysis of immune cells in tumor tissues treated with different nanoaccines. (A) Representative immunohistochemical images showing infiltrating CD8^+^ cells and Foxp3^+^ cells in the tumor tissue. Blue: nuclei; green: CD8^+^ regions; red: Foxp3 regions. Scale bar: 20 μm. (B) Histological evaluation and TUNEL assay in the tumor. Blue: nuclei; green: TUNEL-positive regions. Scale bar: 20 μm. (For interpretation of the references to color in this figure legend, the reader is referred to the Web version of this article.)

In the tumor tissues, treatment with the Mg^2+^-adjuvanted LNP vaccine resulted in a significant increase in the infiltration of CD8^+^ T cells, or cytotoxic T lymphocytes (CTLs), a critical component for killing tumor cells (Fig. 6A and B). This effect was accompanied by a slight reduction in myeloid-derived suppressor cells (MDSCs) and regulatory T cells (Tregs), known for their role in suppressing antitumor immunity (Fig. 6C and D). IHC analysis of the tumor tissue corroborated these findings, showing enhanced CTL infiltration and diminished Treg presence. The TUNEL assay further aligned with flow cytometry results, confirming increased tumor cell apoptosis, which indicates enhanced tumor cell clearance following vaccination (Fig. 7A and B). Together, these data suggest that Mg^2+^ facilitates an immunologically favorable tumor microenvironment by supporting CTL recruitment and reducing immunosuppressive Treg presence, thereby enhancing antitumor responses.

**Fig. 8:**
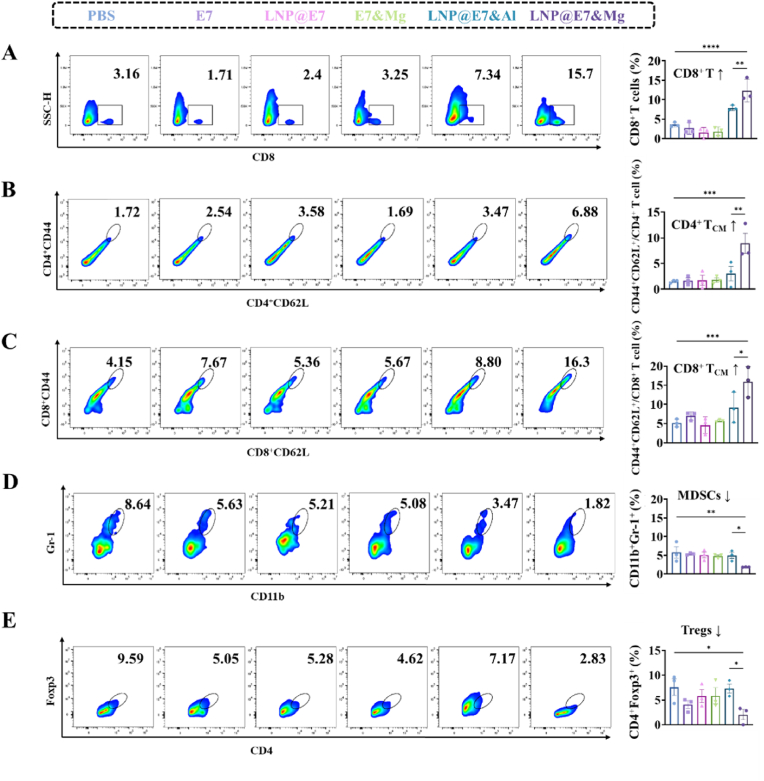
Splenic immune response assay induced by nanovaccines. (A) Flow cytometry analysis and statistical data of splenic CD8^+^ T cells; (B, C) Representative flow cytometry plots and statistical data of splenic CD4^+^ and CD8^+^ central memory T cells (T_CM_); (D) Representative flow cytometry plots and statistical data of splenic MDSCs (CD11b^+^Gr-1^+^); (E) Representative flow cytometry plots and statistical data of the splenic Tregs (CD4^+^Foxp3^+^).∗p < 0.05, ∗∗p < 0.01, ∗∗∗p < 0.001, ∗∗∗∗p < 0.0001, was considered significantly different between compared groups using ANOVA test.

In the spleen, an essential secondary lymphoid organ, the Mg^2+^-adjuvanted LNP vaccine induced notable increases in CD8^+^ T cells, indicating enhanced T cell proliferation and activation compared to controls (Fig. 8A). This suggests that Mg^2+^ enhances the generation of activated CD8^+^ T cells, which can mobilize to tumor sites to exert cytotoxic effects. Additionally, there was a marked induction of central memory T cells (T_CM_), including both CD4^+^CD44^+^CD62L^+^ and CD8^+^CD44^+^CD62L^+^ populations (Fig. 8B and C). Central memory T cells are essential for long-term immune surveillance, as they retain antigen-specific memory, enabling rapid responses upon subsequent encounters with the same antigen [44]. The sustained increase in these memory cells demonstrates that the Mg^2+^ adjuvant supports durable immune memory, potentially contributing to long-lasting antitumor immunity. Furthermore, Mg^2+^ treatment reduced the accumulation of splenic MDSCs (CD11b^+^Gr-1^+^) and Tregs (CD4^+^Foxp3^+^) (Fig. 8D and E). These immunosuppressive cells are known to inhibit T cell responses, and their reduction suggests a lower systemic immunosuppressive burden, potentially contributing to the robust antitumor immunity observed with Mg^2+^ adjuvantation.

**Fig. 9:**
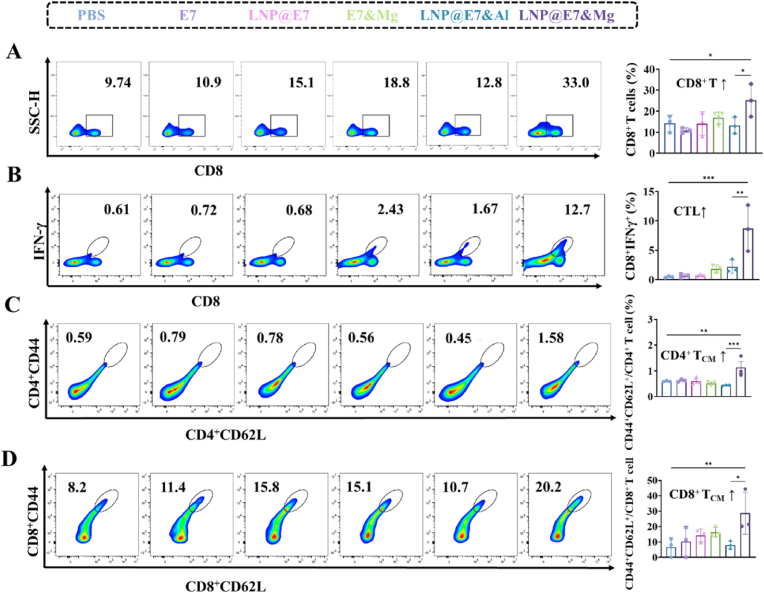
Immune response assay in lymph nodes induced by nanovaccines. (A, B) Flow cytometry analysis and statistical data of CD8^+^ T cell and CTLs content in the lymph nodes of mice; (C, D) Representative flow cytometry plots and statistical data of CD8^+^ and CD4^+^ central memory T cells (T_CM_) in the lymph nodes. ∗p < 0.05, ∗∗p < 0.01, ∗∗∗p < 0.001, was considered significantly different between compared groups using ANOVA test.

Lymph nodes, as key sites for immune cell activation and differentiation, directly drain immune cells to the tumor and reflect local immune activation [45]. In the draining lymph node, the Mg^2+^-adjuvanted LNP vaccine elicited significant increases in activated CD8^+^ T cells and central memory T cell populations, mirroring the effects seen in the spleen (Fig. 9A–D). This proliferation of effector and memory T cells suggests that Mg^2+^ not only promotes immune activation at the tumor site but also drives systemic immune readiness, enabling rapid mobilization of immune cells to the tumor upon antigen recognition. Additionally, the observed reduction in the average levels of MDSCs and Tregs in lymph nodes indicates a local immunological shift towards reduced immune suppression, further supporting an effective antitumor response (Figs. S15A–B).

The Mg^2+^-adjuvanted LNP nanovaccine effectively alters both local and systemic immune landscapes by enhancing CTL infiltration in the tumor microenvironment, reducing immunosuppressive cell populations in the spleen and lymph nodes, and promoting T_CM_ development. Together, these modulations suggest that Mg^2+^ augments antitumor efficacy through comprehensive immune activation and suppression of immune-regulatory pathways. IHC and TUNEL assay results are consistent with flow cytometry findings, collectively underscoring the pivotal role Mg^2+^ in reshaping the immune microenvironment to favor tumor immunity.

In recent years, numerous adjuvants have been developed for cancer immunotherapy, including inorganic material-based adjuvants such as Mg, Zn, Si, and Al [46], hybrid adjuvants like coordination polymers [47,48], and TLR agonist-nanomaterial composites [49]. Among these, magnesium (Mg) stands out for its biocompatibility and unique immunomodulatory effects, including enhanced dendritic cell activation and the promotion of Th1-polarized immune responses, critical for cancer immunotherapy. Unlike other inorganic adjuvants, such as manganese (Mn), Mg has distinct immunological properties. While Mn has been extensively validated to activate the STING pathway to achieve immune activation for cancer vaccination [50], Mg does not rely on the same signaling pathways but instead modulates the collagen-CD36 axis for enhancing DC maturation and antigen presentation. Moreover, Mg is generally less toxic than Mn [51], while high concentrations of Mn can lead to neurotoxicity and other health risks, Mg is essential for physiological processes and poses minimal toxicity, making it a safer option for therapeutic use, which makes it a potentially safer and more versatile adjuvant in the context of cancer immunotherapy. The advantage of Mg-based LNP formulations lie in their simplicity, tunability, and reduced toxicity compared to more complex hybrid or TLR agonist-based systems, making them an attractive option for scalable and efficient cancer immunotherapy development.

### 3.7 Biosafety of LNP vaccines

Five physiological and biochemical indicators (AST, ALT, CK, CR and BUN) in the blood of mice from each group were evaluated, the results showed that the values of mice treated with LNP@E7&Mg were within the normal range (Fig. S16A). H&E staining of major organs (heart, liver, spleen, kidney, and lung) from mice treated with different drugs (Fig. S16B) were performed. The results showed that, compared to the tissue morphology of mice treated with PBS, there were no significant morphological changes in the major organs of mice treated with LNP@E7&Mg. These data collectively confirm good biosafety profile of the LNP@E7&Mg vaccine.

## 4 Conclusions

This study highlights the potential of magnesium as a novel adjuvant in LNP-based therapeutic vaccines for HPV-associated cancers. By leveraging microfluidic technology, an optimized LNP formulation was developed to co-load the E7 antigen and magnesium ions, achieving effective delivery with enhanced immune activation. Unlike traditional aluminum-based adjuvants, magnesium ions demonstrated a distinct capacity to promote T-cell responses and immunity, addressing limitations of conventional adjuvants in cancer immunotherapy. Mechanistic investigation suggest that magnesium ions may enhance DC maturation and antigen presentation through a collagen-CD36 axis, evidenced by upregulated collagen-related gene expression and collagen chain trimerization.

This research provides a foundation for further investigation of magnesium as an adjuvant, with implications for broader applications in therapeutic cancer vaccines. As the LNP@E7&Mg formulation advances through preclinical stages, its design and effectiveness could support clinical translation, offering a promising new strategy for enhancing the efficacy and safety of mucosal vaccines targeting HPV and potentially other cancers. The results underscore the value of continued exploration into microfluidic-assisted LNP systems and magnesium-based adjuvants, paving the way for innovative approaches in cancer immunotherapy.

## CRediT authorship contribution statement

**Yongyi Xie:** Writing – original draft, Methodology, Data curation. **Jiaxin Guo:** Methodology, Data curation. **Jialin Hu:** Methodology, Data curation. **Yuan Li:** Resources, Data curation. **Zhongqian Zhang:** Resources, Data curation. **Yongcheng Zhu:** Writing – review & editing. **Fei Deng:** Writing – review & editing. **Jialong Qi:** Writing – review & editing, Supervision, Conceptualization. **You Zhou:** Writing – review & editing, Supervision, Conceptualization. **Wenjie Chen:** Writing – review & editing, Supervision, Funding acquisition, Conceptualization.

## Declaration of competing interest

The authors declare that they have no known competing financial interests or personal relationships that could have appeared to influence the work reported in this paper.

## Data Availability

Data will be made available on request.
