# Supplementary material for: A factorial design-optimized microfluidic LNP vaccine elicits potent magnesium-adjuvating cancer immunotherapy
Source: Mater Today Bio. 2025 Mar 24;32:101703. doi: 10.1016/j.mtbio.2025.101703 (PMC11994397; doi:10.1016/j.mtbio.2025.101703)
Supplement: Multimedia component 1 [file mmc1.docx]

**Supplementary information:**

**A Factorial Design-optimized Microfluidic LNP Vaccine Elicits Potent Magnesium-Adjuvating Cancer Immunotherapy**

Yongyi Xie^1,5^, Jiaxin Guo^1,5^, Jialin Hu^1,5^, Yuan Li^1^, Zhongqian Zhang^1^, Yongcheng Zhu^2^, Fei Deng^4^, Jialong Qi^3*^, You Zhou^1*^, Wenjie Chen^1*^

^1^Guangdong Province & NMPA & State Key Laboratory, School of Pharmaceutical Sciences; Guangzhou Medical University, Guangzhou, 511436, P.R. China.

^2^Department of Emergency, The Second Affiliated Hospital, Guangzhou Medical University, Guangzhou, 510260, P.R. China.

^3^Yunnan Digestive Endoscopy Clinical Medical Center, Department of Gastroenterology, The First People's Hospital of Yunnan Province, Affiliated Hospital of Kunming University of Science and Technology, Kunming 650032, P.R. China.

^4^Graduate School of Biomedical Engineering, ARC Centre of Excellence in Nanoscale Biophotonics, Faculty of Engineering, UNSW Sydney, NSW 2052, Australia.

^5^Authors contribute equally to this work

**Corresponding author:**

W. Chen: wjchen@gzhmu.edu.cn, https://orcid.org/0000-0001-9512-9664

J. Qi: qijialong1989@imbcams.com.cn, https://orcid.org/0000-0001-6361-7479

Y. Zhou: zyou@gzhmu.edu.cn, https://orcid.org/0000-0002-0779-9519

Table S1. DSD design (3^3^) and the experimental runs for optimizing the conditions of different total lipid concentrations, FRR, and TFR in LNP preparation.

| Run no. | Lipid conc. | FRR | TFR | Size | PDI |
| --- | --- | --- | --- | --- | --- |
| 1 | 2.5 | 8 | 3.2 | 132 | 0.337 |
| 2 | 2.5 | 2 | 1.2 | 476 | 0.125 |
| 3 | 4 | 5 | 1.2 | 134.7 | 0.227 |
| 4 | 1 | 5 | 3.2 | 202.2 | 0.258 |
| 5 | 4 | 2 | 2.2 | 169.6 | 0.247 |
| 6 | 1 | 8 | 2.2 | 129.6 | 0.311 |
| 7 | 4 | 8 | 1.2 | 353.3 | 0.115 |
| 8 | 1 | 2 | 3.2 | 128 | 0.141 |
| 9 | 4 | 8 | 3.2 | 135.6 | 0.333 |
| 10 | 1 | 2 | 1.2 | 177.3 | 0.073 |
| 11 | 4 | 2 | 3.2 | 151.5 | 0.123 |
| 12 | 1 | 8 | 1.2 | 123.1 | 0.103 |
| 13 | 2.5 | 5 | 2.2 | 274.8 | 0.125 |
| 14 | 2.5 | 5 | 2.2 | 122.3 | 0.135 |

Table S2. Factorial design (3×3×3, 1-27) for screening lipid type, buffer system, and E7 dosage.

| Run no | Lipid | Buffer | E7 | Size (nm) | PDI | EE% |
| --- | --- | --- | --- | --- | --- | --- |
| 1 | DOTAP | HEPES (25mM) | 0.05 | 167.4 | 0.309 | 94.15 |
| 2 | DOTAP | HEPES (25mM) | 0.1 | 253 | 0.373 | 76.88 |
| 3 | DOTAP | HEPES (25mM) | 0.2 | 231.8 | 0.404 | 83.48 |
| 4 | DOTAP | DEPC (0.1%) | 0.05 | 101.1 | 0.402 | 96.55 |
| 5 | DOTAP | DEPC (0.1%) | 0.1 | 124.4 | 0.377 | 90.27 |
| 6 | DOTAP | DEPC (0.1%) | 0.2 | 114.2 | 0.306 | 89.51 |
| 7 | DOTAP | citric acid | 0.05 | 157.6 | 0.252 | 79.54 |
| 8 | DOTAP | citric acid | 0.1 | 204.82 | 0.525 | 87.69 |
| 9 | DOTAP | citric acid | 0.2 | 154.3 | 0.296 | 72.21 |
| 10 | DODAP | HEPES (25mM) | 0.05 | 178.2 | 0.388 | 76.19 |
| 11 | DODAP | HEPES (25mM) | 0.1 | 196.8 | 0.381 | 58 |
| 12 | DODAP | HEPES (25mM) | 0.2 | 228.3 | 0.422 | 46.4 |
| 13 | DODAP | DEPC (0.1%) | 0.05 | 127.27 | 0.351 | 41.98 |
| 14 | DODAP | DEPC (0.1%) | 0.1 | 134.5 | 0.353 | 78.4 |
| 15 | DODAP | DEPC (0.1%) | 0.2 | 125.5 | 0.351 | 75.09 |
| 16 | DODAP | citric acid | 0.05 | 143 | 0.34 | 81.7 |
| 17 | DODAP | citric acid | 0.1 | 167.4 | 0.342 | 75.79 |
| 18 | DODAP | citric acid | 0.2 | 184.6 | 0.372 | 62.02 |
| 19 | Dlin-MC3 | HEPES (25mM) | 0.05 | 199.4 | 0.451 | 72.1 |
| 20 | Dlin-MC3 | HEPES (25mM) | 0.1 | 169.6 | 0.394 | 68.54 |
| 21 | Dlin-MC3 | HEPES (25mM) | 0.2 | 231.9 | 0.35 | 71.71 |
| 22 | Dlin-MC3 | DEPC (0.1%) | 0.05 | 121.9 | 0.321 | 88.97 |
| 23 | Dlin-MC3 | DEPC (0.1%) | 0.1 | 153.9 | 0.356 | 82.92 |
| 24 | Dlin-MC3 | DEPC (0.1%) | 0.2 | 120.2 | 0.191 | 56.72 |
| 25 | Dlin-MC3 | citric acid | 0.05 | 242.9 | 0.637 | 0.00 |
| 26 | Dlin-MC3 | citric acid | 0.1 | 294.8 | 0.658 | 82.4 |
| 27 | Dlin-MC3 | citric acid | 0.2 | 449.4 | 0.575 | 67.53 |

Table S3. Drug loading capacity (LC) and encapsulation efficiency (EE) data of E7 and Mg^2+^ in the LNP Vaccines.

|  | LC of E7 (%) | EE of E7 (%) | LC of Mg^2+^ (%) | EE of Mg^2+^ (%) |
| --- | --- | --- | --- | --- |
|  | 1.28±0.06 | 89.51±0.4 | 2.59±0.3 | 15.52±0.1 |

Table S4 Antibodies used in flow cytometry.

| Name of antibody | Manufacter | Catalogue number |
| --- | --- | --- |
| PE-CD8a | Elabscience | F1104D |
| PE-CD80 | Elabscience | F0992D |
| APC-CD86 | Elabscience | F0994E |
| FITC-CD4 | Elabscience | F1097C |
| FITC-CD11b | Elabscience | F1081C |
| APC-IFN-γ | Elabscience | F1101E |
| PE-Foxp3 | Elabscience | F1238D |
| PE-Gr-1 | Elabscience | F1120D |
| APC-CD44 | Elabscience | F1100E |
| PerCP/Cyanine5.5-CD62L | Elabscience | F1011J |
| PerCP/Cyanine5.5-CD3 | Elabscience | F1013J |


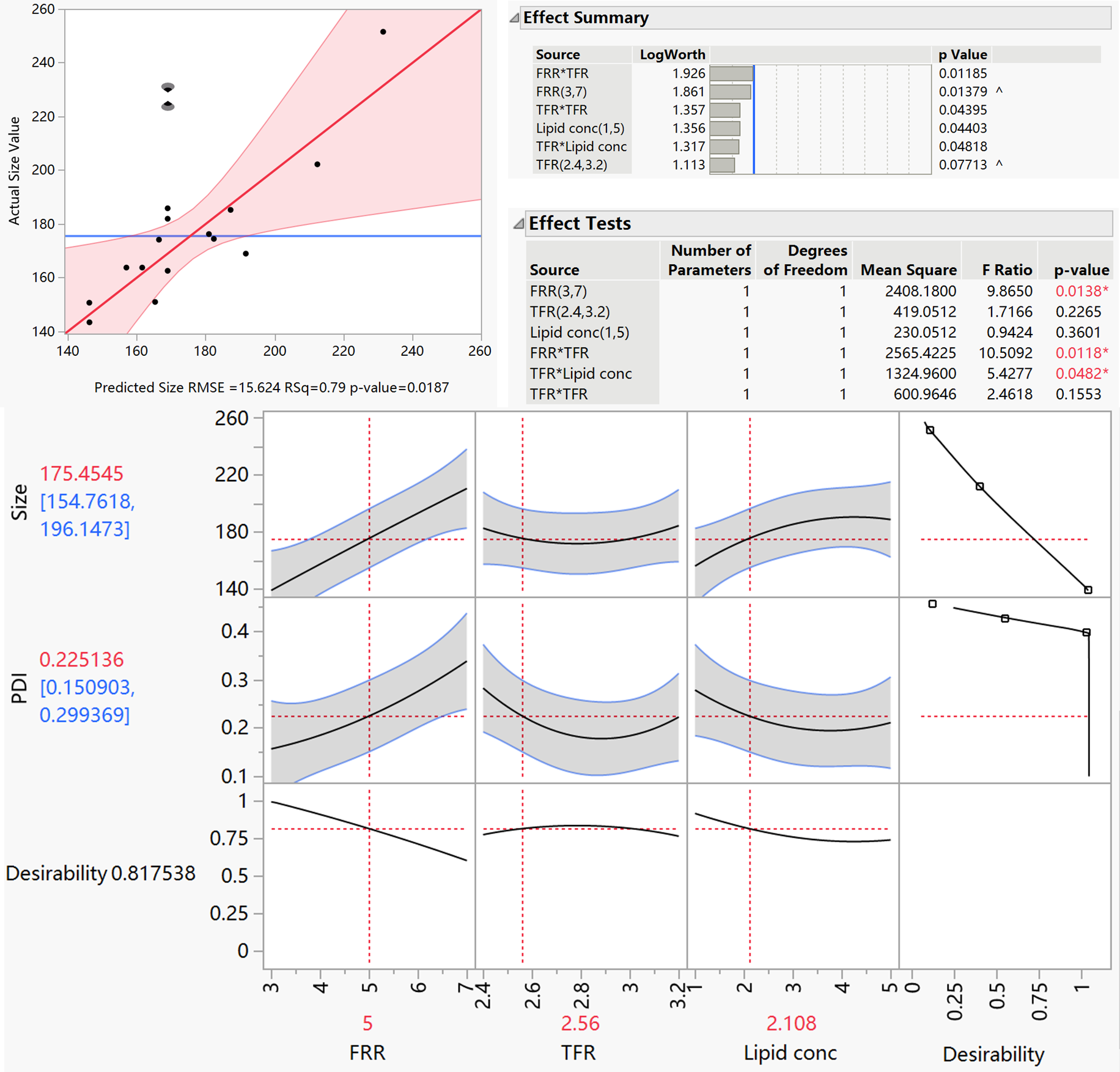


Figure S1. The analysis results using JMP Pro software with the Box-Behnken DoE, exploring the interactions and effects of FRR, TFR, and lipid concentration on blank LNP particle size and PDI.


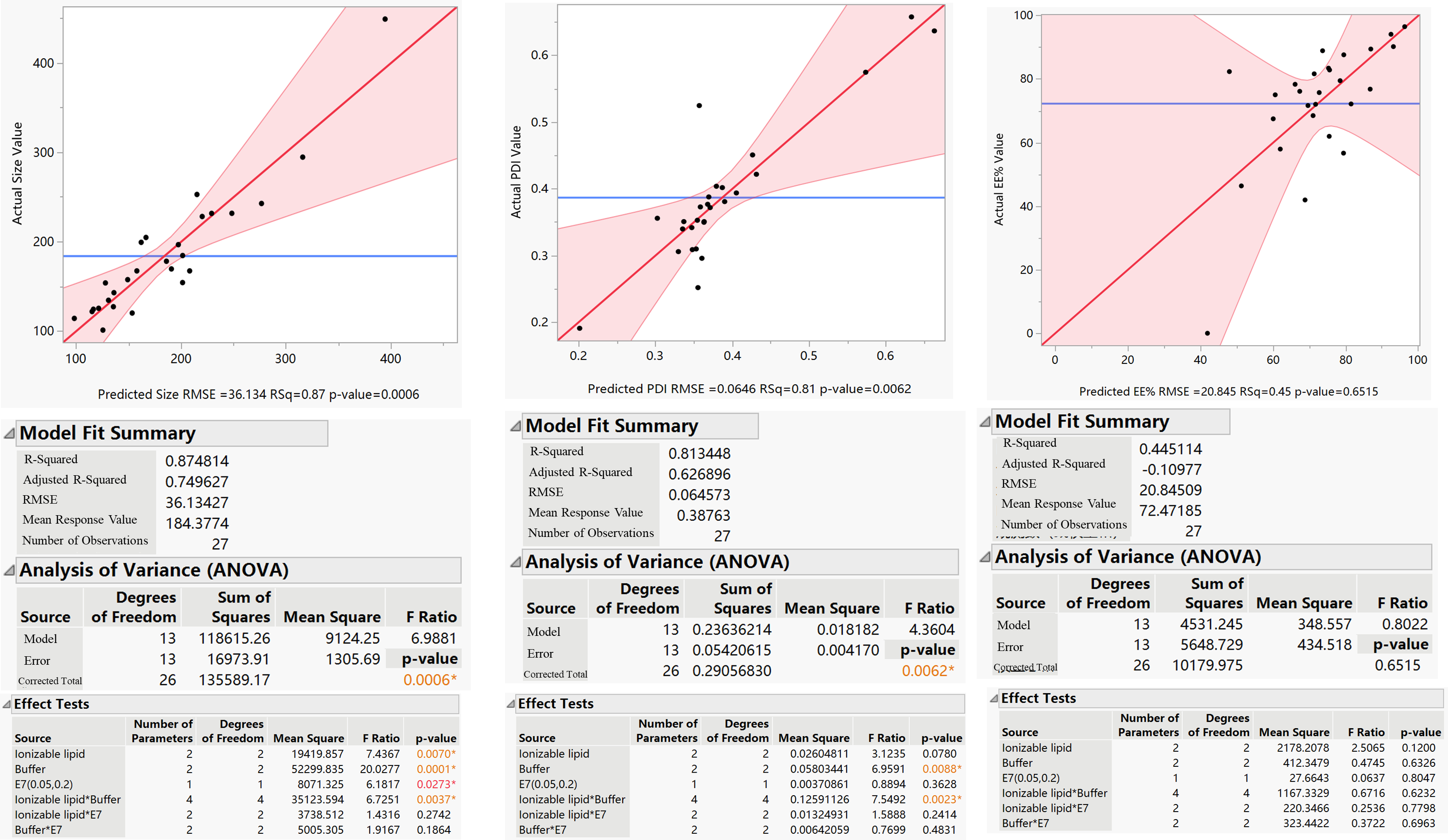


Figure S2. Presents the data analysis using JMP Pro software with factorial design, evaluating the main effects and interactions of lipid composition, buffer type, and E7 concentration on the response values (particle size, PDI, and encapsulation efficiency (EE%)) in preparing LNP@E7.


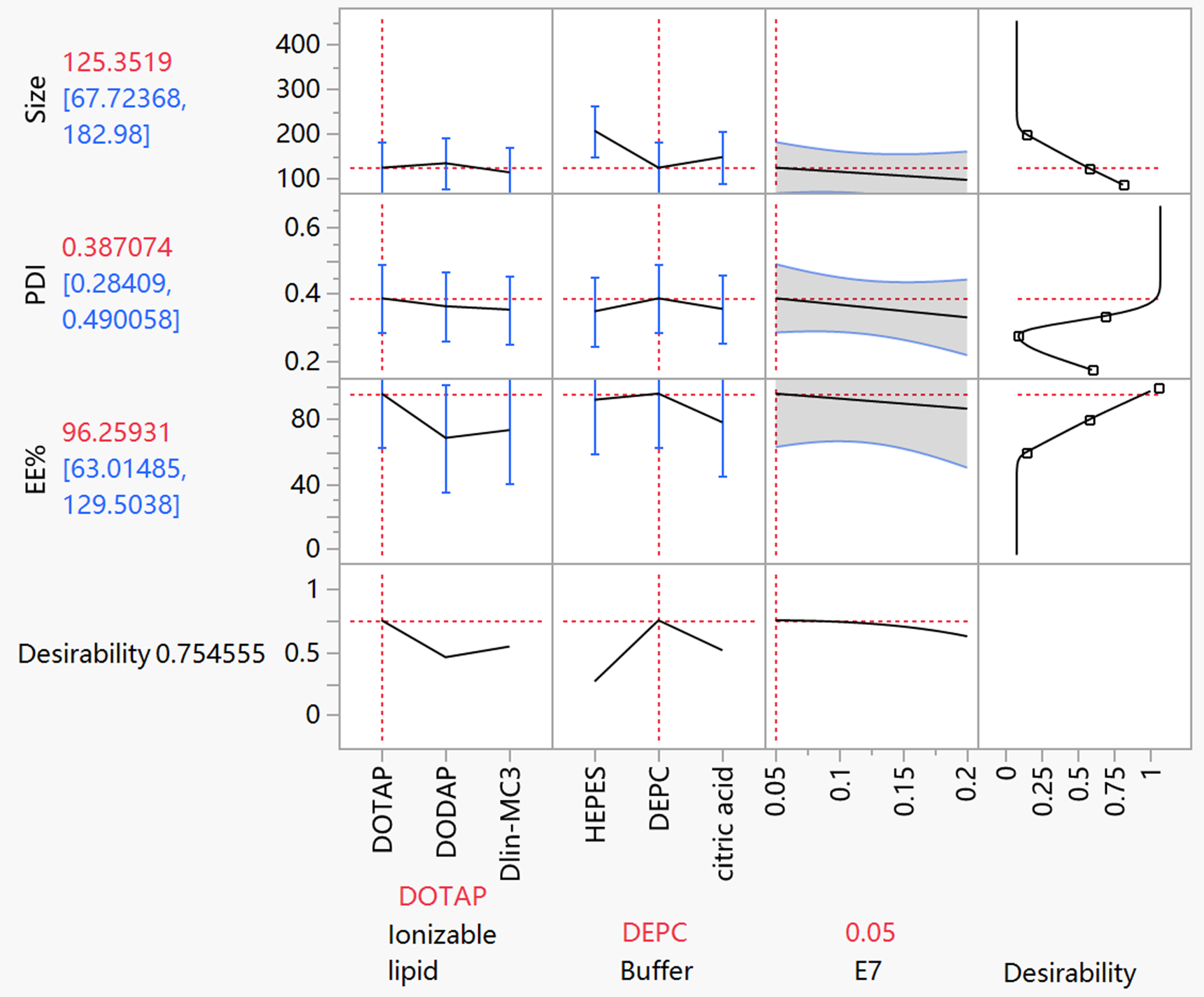


Figure S3. The data analysis using JMP Pro software with factorial design, predicting the influence of various factors on the LNP@E7 response values (particle size (Size), PDI, and encapsulation efficiency (EE%)).


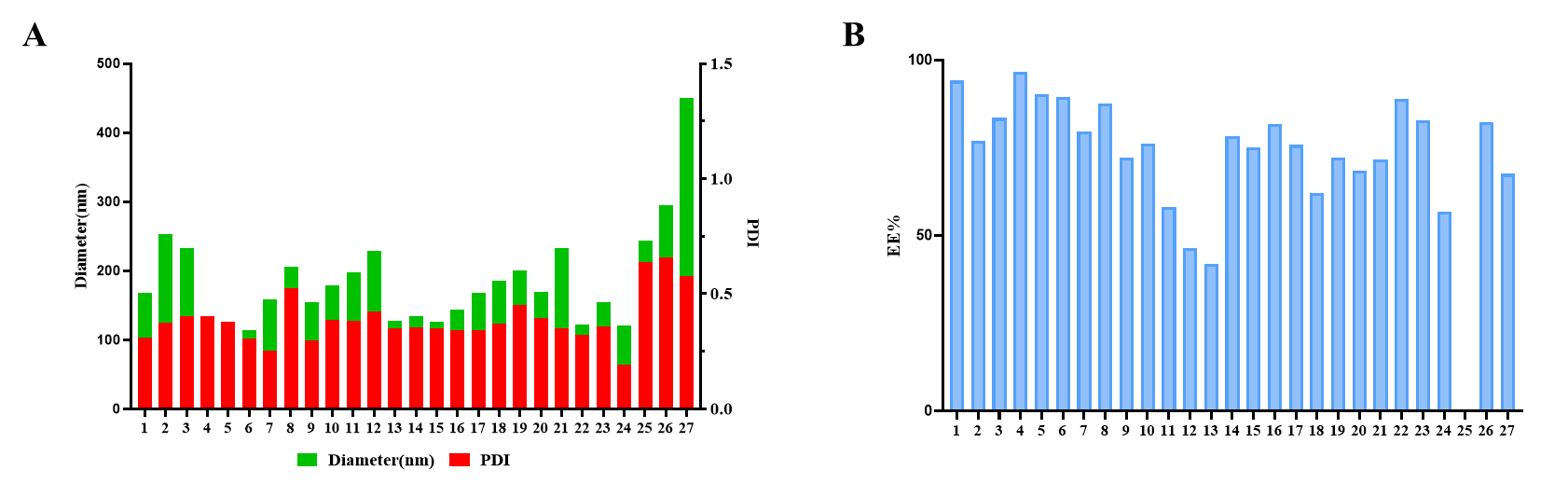


Figure S4. Optimization of the LNP@E7 System. (A) The average particle size of the LNP-E7 system and polydispersity index (PDI) of the LNP@E7 system; (B) Encapsulation efficiency (EE%) of the LNP@E7 system.

Figure S5. The release curves of Mg^2+^ and E7 in LNP@Mg&E7 in PBS over 72 hours.


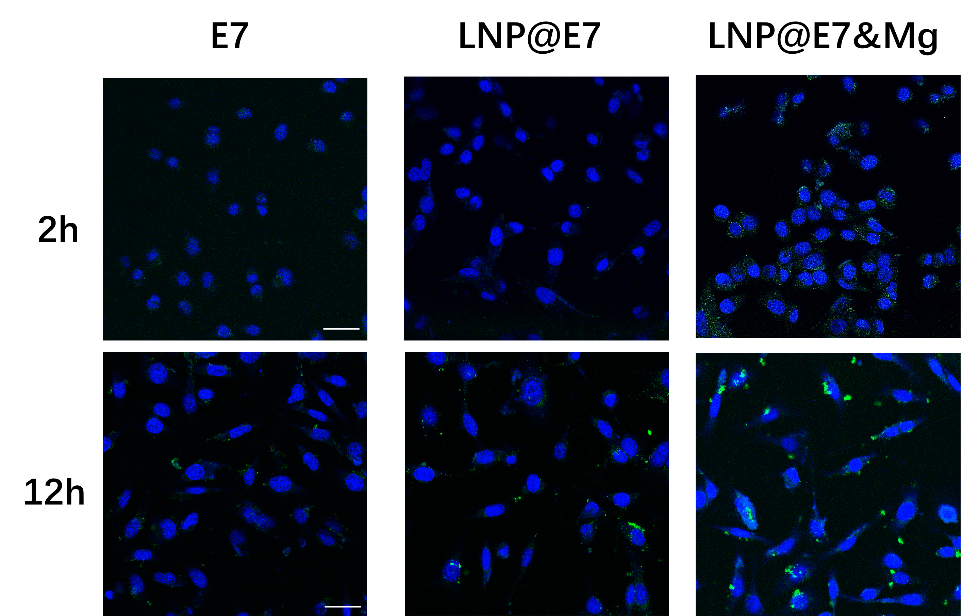


Figure S6. Confocal microscopy images of DC2.4 cells showing the cellular uptake of E7 peptide after 2 hours and 12 hours of incubation, scale bar: 50μm.


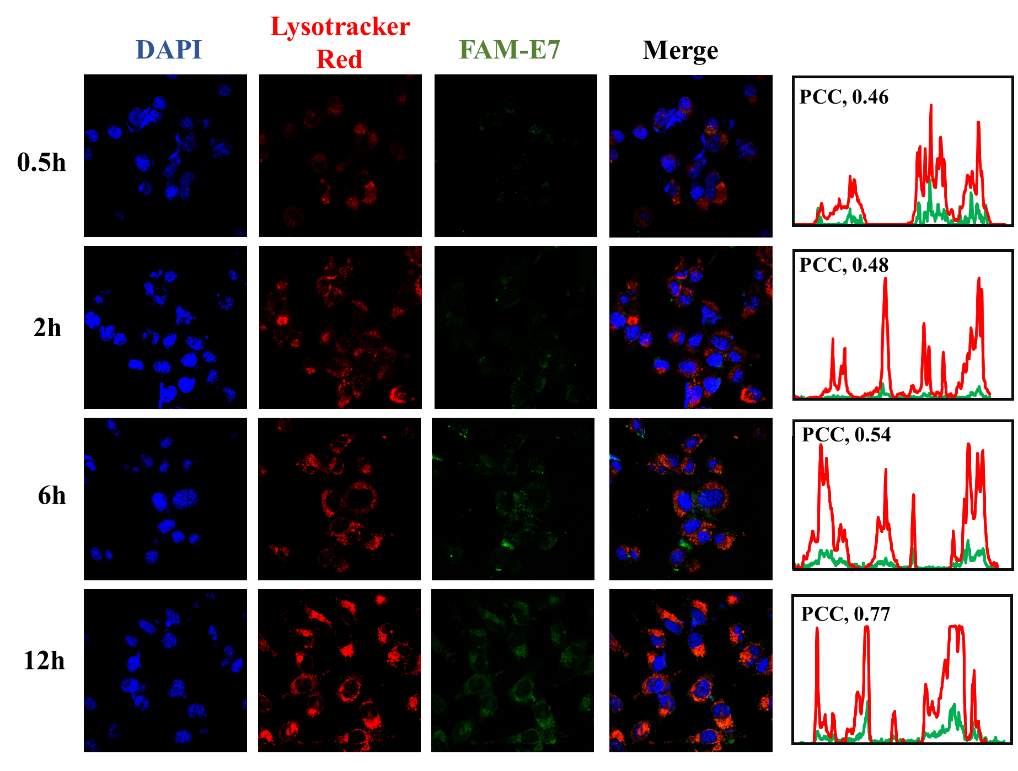


Figure S7. The CLSM images showing lysosomal escape of E7 and the corresponding co-localization analysis E7-FAM at 0.5h, 2h, 6h, and 12h.


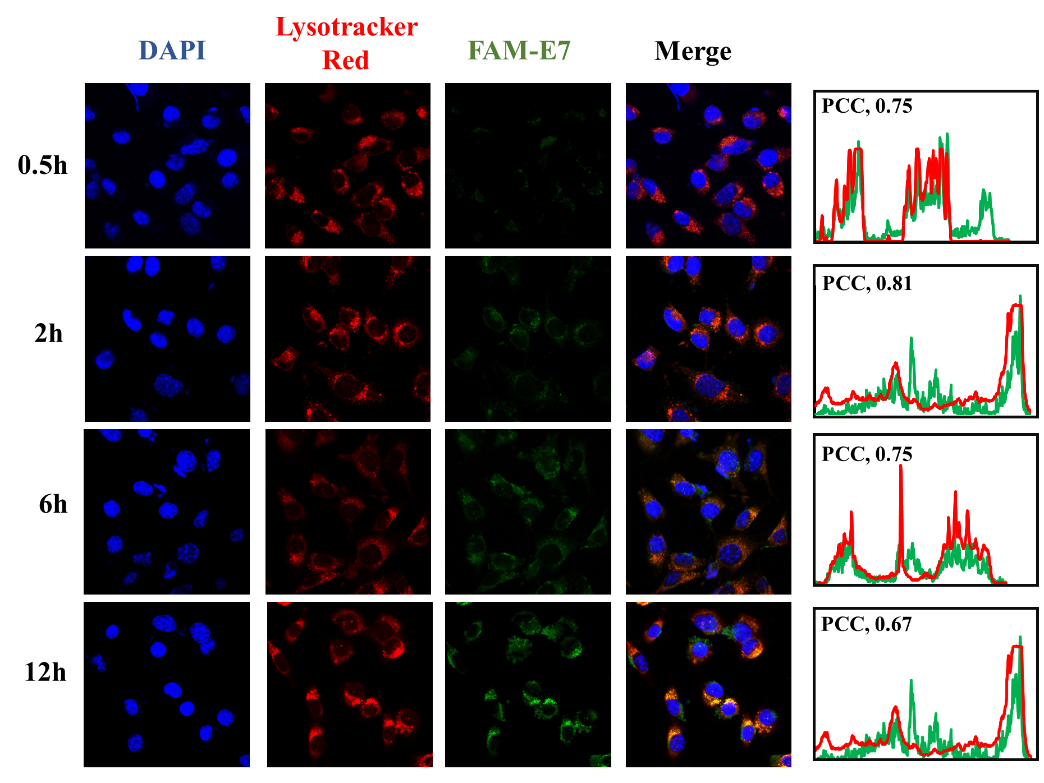


Figure S8. The CLSM images showing lysosomal escape of E7 and the corresponding co-localization analysis of LNP@E7-FAM at 0.5h, 2h, 6h, and 12h.


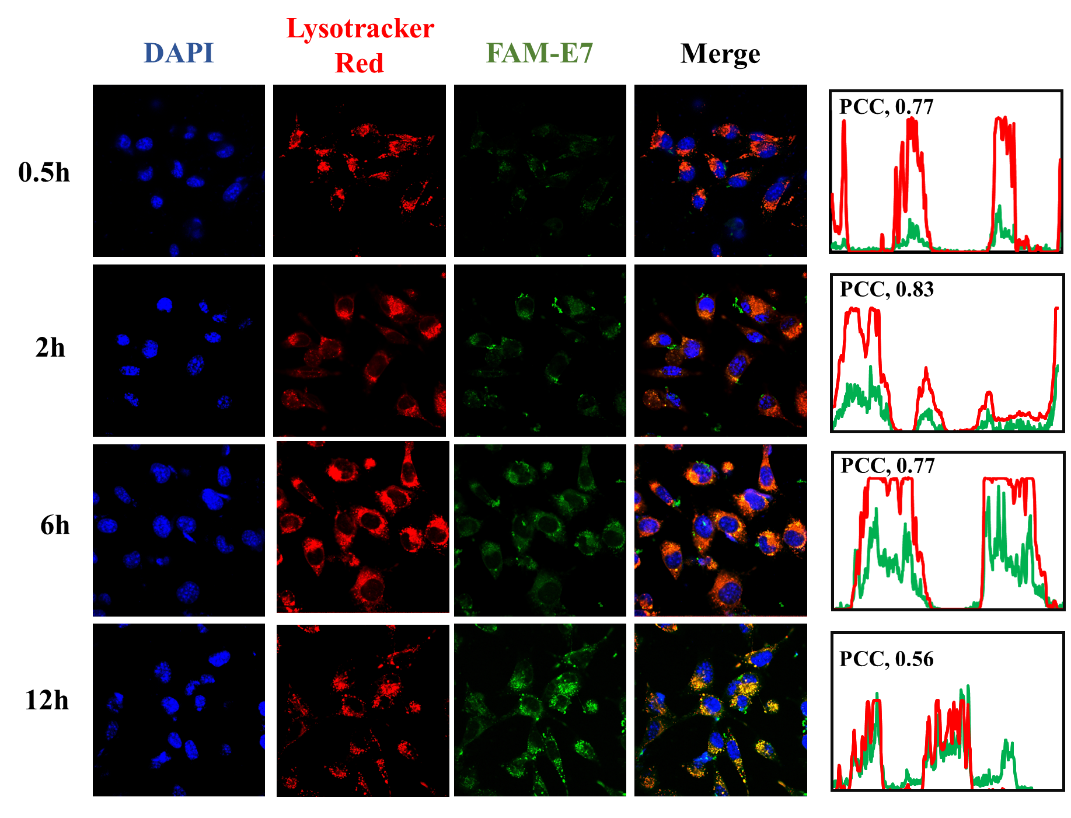


Figure S9 The CLSM images showing lysosomal escape of E7 and the corresponding co-localization analysis of LNP@E7-FAM&Mg at 0.5h, 2h, 6h, and 12h.


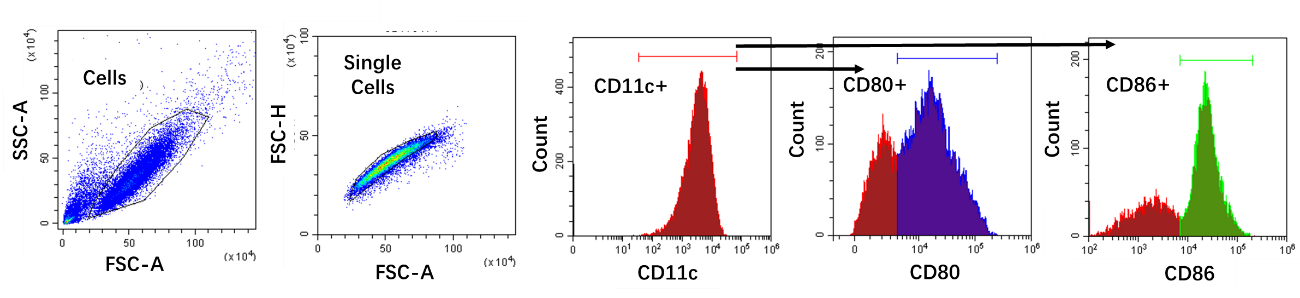


Figure S10. The gating strategy of BMDCs maturation (CD80^+^ and CD86^+^).


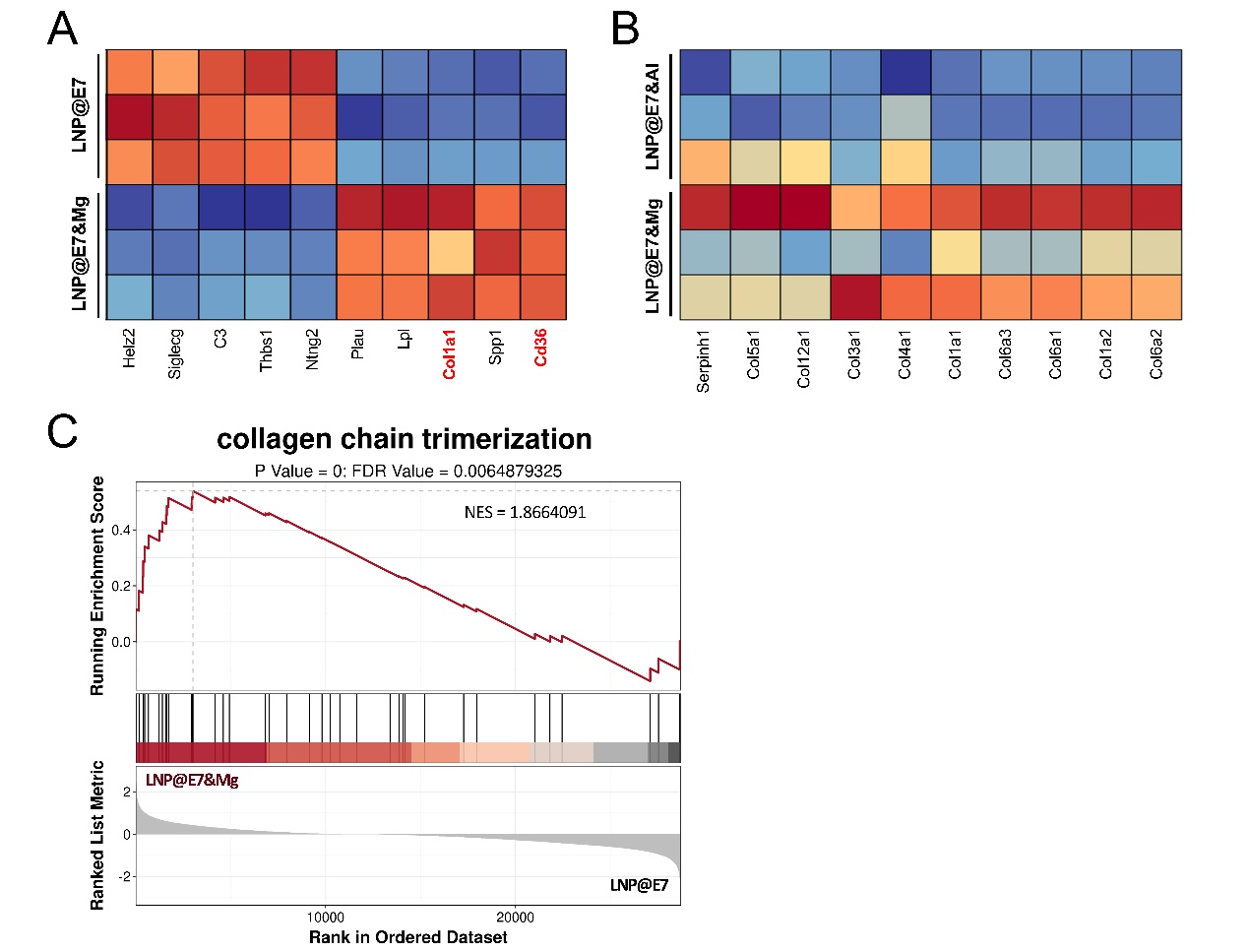
Figure S11. (A) The top differently expressed genes in LNP@E7&Mg and LNP@E7 treated BMDCs; (B) The expression pattern of representative collagen formation genes in LNP@E7&Mg and LNP@E7&Al treated BMDCs; (C) GSEA analyzed enrichment of collagen chain trimerization for BMDCs treated with LNP@E7&Mg and LNP@E7.


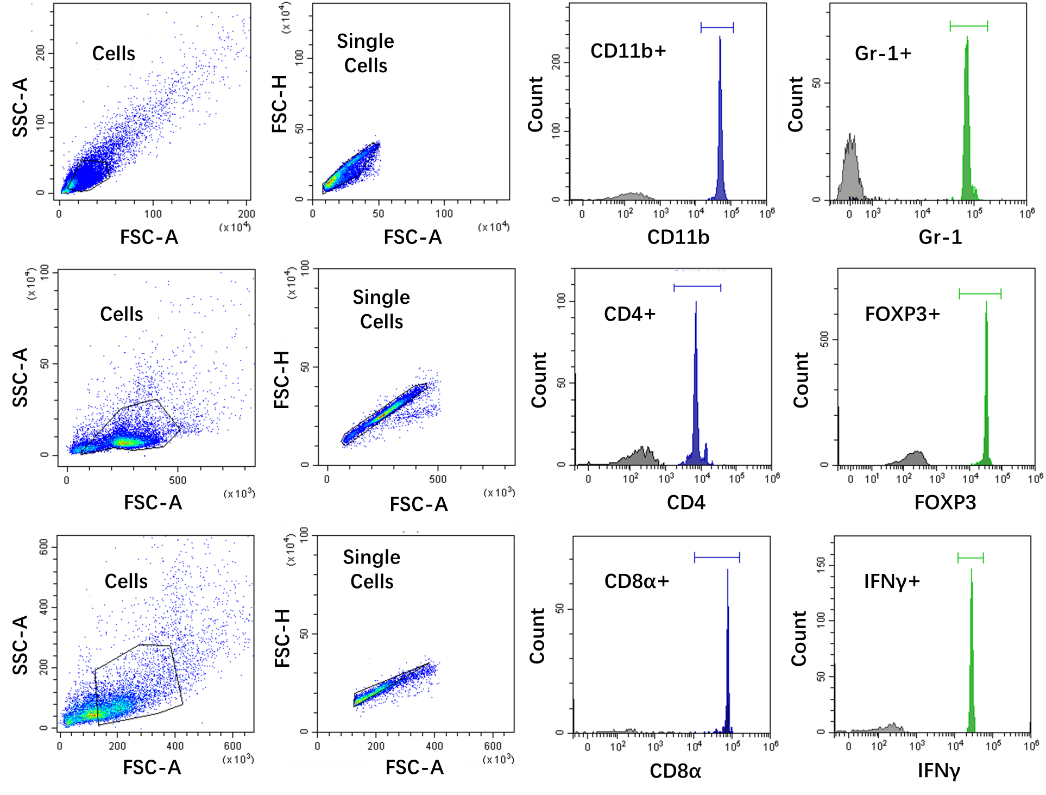


Figure S12. The gating of mice tumor immune cells (MDSCs, Tregs, CTL from top to bottle row).


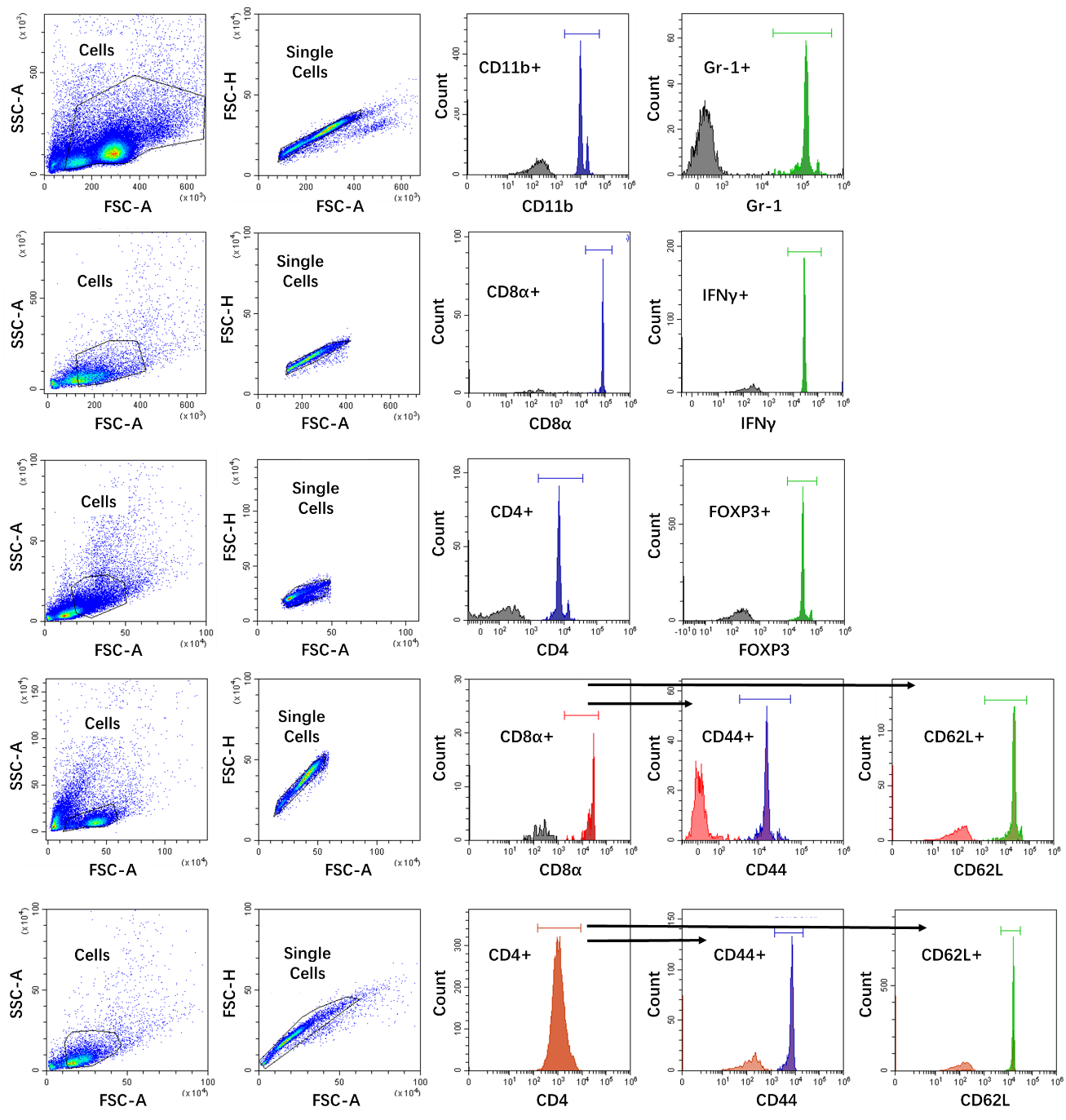


Figure S13. The gating strategies of mice splenic immune cells (MDSCs, CTL, Tregs, CD8^+^ T_CM_, CD4 ^+^ T_CM_, from top to bottle row).


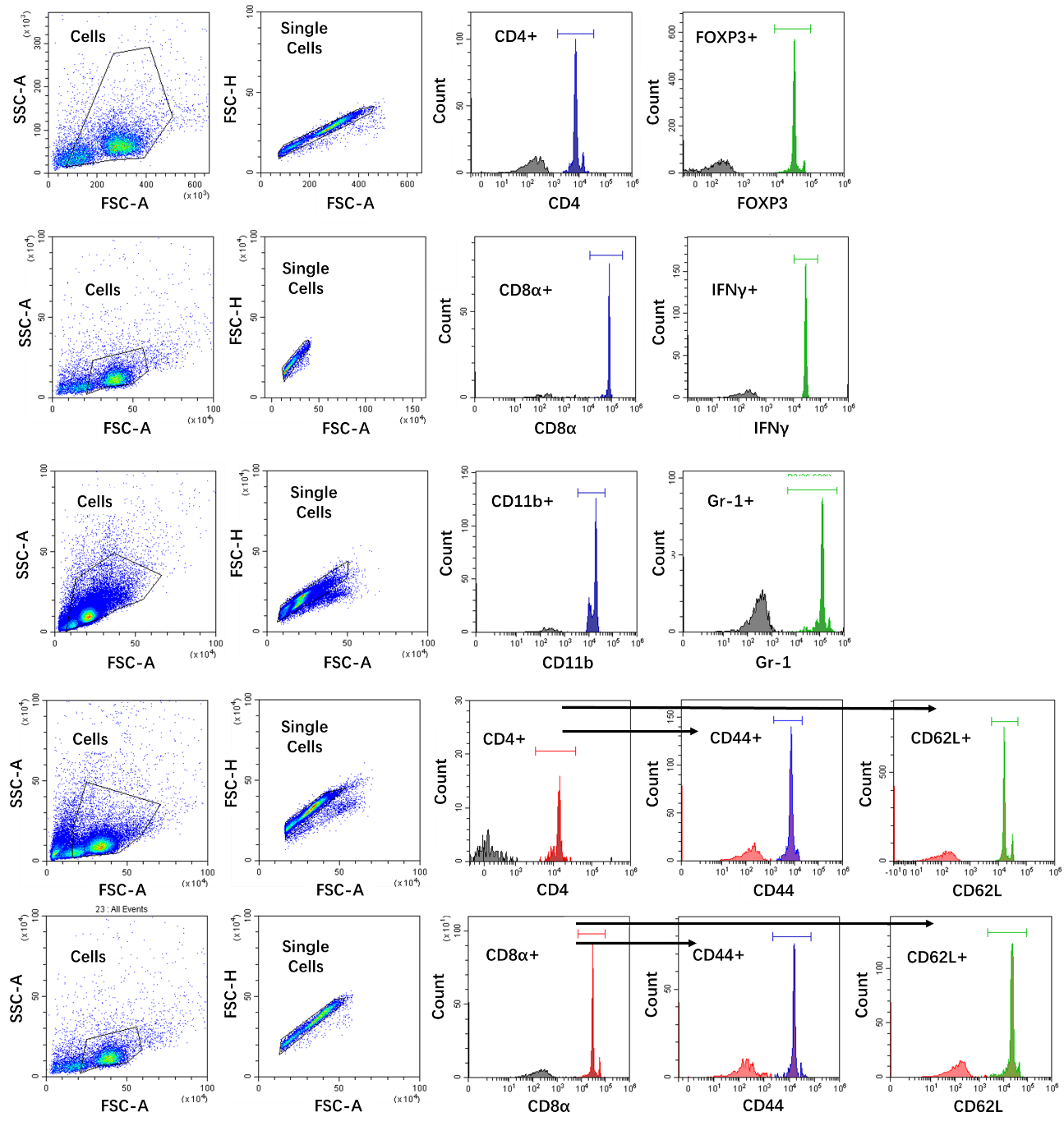


Figure S14. The gating strategies of mice lymph nodes immune cells (Tregs, CTL, MDSCs, CD4^+^ T_CM_, CD8^+^ T_CM_, from top to bottle row).


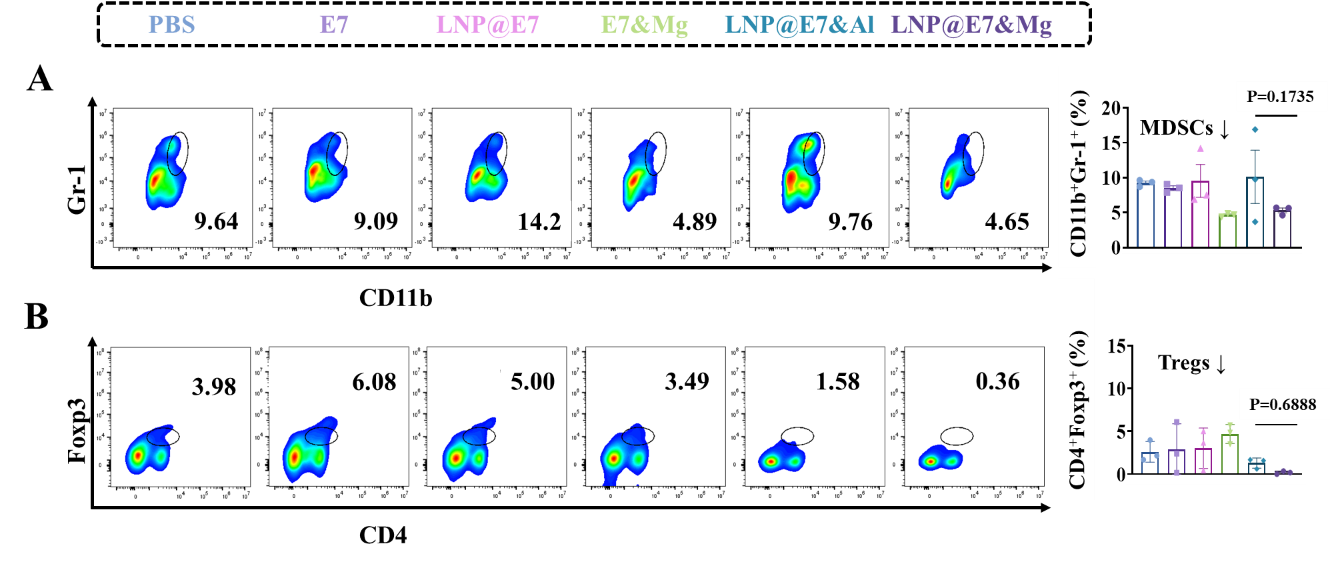


Figure S15. Immune response assay in lymph nodes induced by nanovaccines. (A) Representative flow cytometry plots and statistical data of MDSCs (CD11b^+^ Gr-1^+^) in the lymph nodes; (B) Representative flow cytometry plots and statistical data of tumor-infiltrating Tregs (CD4^+^ Foxp3^+^) in the lymph nodes.


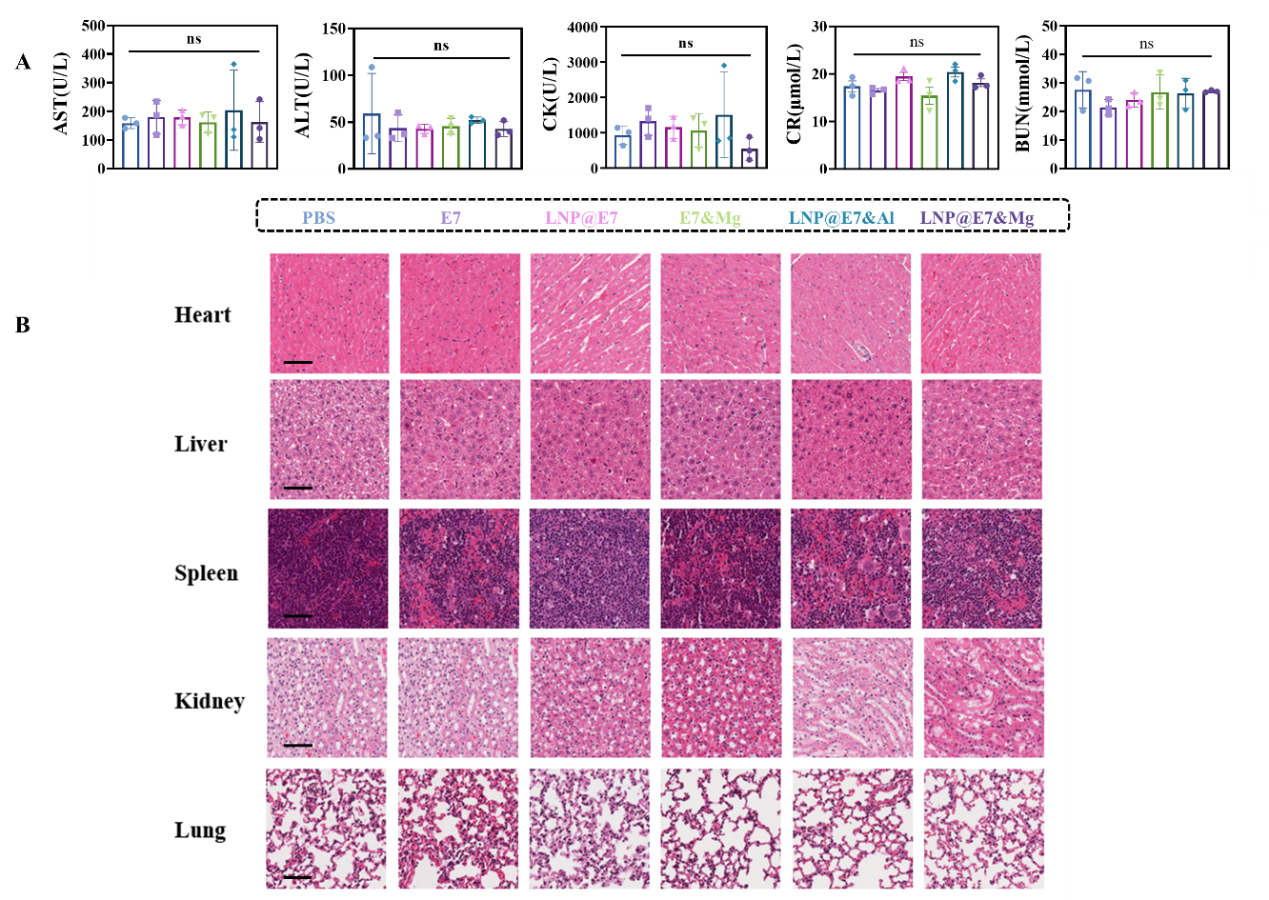


Figure S16. Biosafety assay of LNP vaccines. (A) Statistical chart of serum levels of AST, ALT, CK, CR, and BUN (n = 3); (B) H&E staining of the heart, liver, spleen, lung, and kidney of mice after treatment. Scale bar: 100 μm
